# Accounting for photosystem I photoinhibition sheds new light on seasonal acclimation strategies of boreal conifers

**DOI:** 10.1093/jxb/erae145

**Published:** 2024-04-04

**Authors:** Steffen Grebe, Albert Porcar-Castell, Anu Riikonen, Virpi Paakkarinen, Eva-Mari Aro

**Affiliations:** Molecular Plant Biology, Department of Life Technologies, University of Turku, 20014 Turku, Finland; Optics of Photosynthesis Laboratory, Viikki Plant Science Center, Institute for Atmospheric and Earth System Research/Forest Sciences, University of Helsinki, 00014 Helsinki, Finland; Optics of Photosynthesis Laboratory, Viikki Plant Science Center, Institute for Atmospheric and Earth System Research/Forest Sciences, University of Helsinki, 00014 Helsinki, Finland; Optics of Photosynthesis Laboratory, Viikki Plant Science Center, Institute for Atmospheric and Earth System Research/Forest Sciences, University of Helsinki, 00014 Helsinki, Finland; Molecular Plant Biology, Department of Life Technologies, University of Turku, 20014 Turku, Finland; Molecular Plant Biology, Department of Life Technologies, University of Turku, 20014 Turku, Finland; University of Cambridge, UK

**Keywords:** Conifers, cyclic electron flow, P700 absorbance, photoinhibition, photosystem, *Picea abies*, *Pinus sylvestris*, PSII:PSI stoichiometry, quantum yields, seasonal acclimation

## Abstract

The photosynthetic acclimation of boreal evergreen conifers is controlled by regulatory and photoprotective mechanisms that allow conifers to cope with extreme environmental changes. However, the underlying dynamics of photosystem II (PSII) and photosystem I (PSI) remain unresolved. Here, we investigated the dynamics of PSII and PSI during the spring recovery of photosynthesis in *Pinus sylvestris* and *Picea abies* using a combination of chlorophyll *a* fluorescence, P700 difference absorbance measurements, and quantification of key thylakoid protein abundances. In particular, we derived a new set of PSI quantum yield equations, correcting for the effects of PSI photoinhibition. Using the corrected equations, we found that the seasonal dynamics of PSII and PSI photochemical yields remained largely in balance, despite substantial seasonal changes in the stoichiometry of PSII and PSI core complexes driven by PSI photoinhibition. Similarly, the previously reported seasonal up-regulation of cyclic electron flow was no longer evident, after accounting for PSI photoinhibition. Overall, our results emphasize the importance of considering the dynamics of PSII and PSI to elucidate the seasonal acclimation of photosynthesis in overwintering evergreens. Beyond the scope of conifers, our corrected PSI quantum yields expand the toolkit for future studies aimed at elucidating the dynamic regulation of PSI.

## Teaser

Photosynthetic acclimation in boreal evergreen conifers is characterized by not only photoinhibition of PSII but also of PSI, requiring correction of classical PSI quantum yields to reveal their acclimation strategies.

## Introduction

In northern latitudes, boreal evergreen conifers face large changes in prevailing temperature and light availability throughout the seasons. These environmental cues govern the acclimation response from the whole plant to the molecular level, particularly during winter (Öquist and Huner, 2003; Chang *et al.*, 2021). The acclimation response includes the regulation of photosynthetic reactions, ranging from light harvesting in the photosynthetic antenna complexes (the light harvesting complexes, LHC) and photochemistry catalysed by photosystem I (PSI) and photosystem II (PSII) to CO_2_ assimilation in the Calvin–Benson–Bassham cycle. The coordination of these regulatory responses is essential to maintain the energy balance between the different components of the photosynthetic apparatus, in particular when low temperatures inhibit enzymatic activities but sunlight is still absorbed by leaves of boreal evergreens (Ensminger *et al.*, 2006). Otherwise, overexcitation of photosystems and accumulation of electrons inside the photosynthetic electron transport chain lead to oxidative damage and photoinhibition of PSII (Liu *et al.*, 2019) and PSI (Lima-Melo *et al.*, 2019).

To cope with freezing temperatures and excess light conditions, boreal evergreen conifers dynamically adjust their photosynthetic protein complexes on the seasonal scale (Schöttler and Tóth, 2014; Verhoeven, 2014) and engage in a series of photoprotective mechanisms, including: (i) a combination of reversible and sustained non-photochemical quenching (NPQr and NPQs), preventing the overexcitation of PSII (Demmig-Adams and Adams, 2006; García-Plazaola *et al.*, 2012; Verhoeven, 2014); (ii) rerouting of electrons from linear electron flow (LEF) to alternative electron flow (AEF), which delivers electrons to alternative acceptors not used for CO_2_ assimilation in the Calvin–Benson–Bassham cycle; and (iii) up-regulating cyclic electron flow (CEF), which shuttles electrons around PSI back into the plastoquinone pool. Both AEF and CEF are mediated by various molecular pathways preventing accumulation of electrons inside the electron transport chain and facilitating photoprotective functions under environmental stress conditions (Yamori and Shikanai, 2016; Alric and Johnson, 2017; Alboresi *et al.*, 2019b; Nawrocki *et al.*, 2019). Although previous studies of the seasonal acclimation in boreal evergreen conifers have highlighted the importance of AEF (Savitch *et al.*, 2010; Bag *et al.*, 2023) and CEF (Ivanov *et al.*, 2001; Fréchette *et al.*, 2015; Yang *et al.*, 2020), the underlying dynamics of PSI and PSII remain unresolved.

**Fig. 1.**
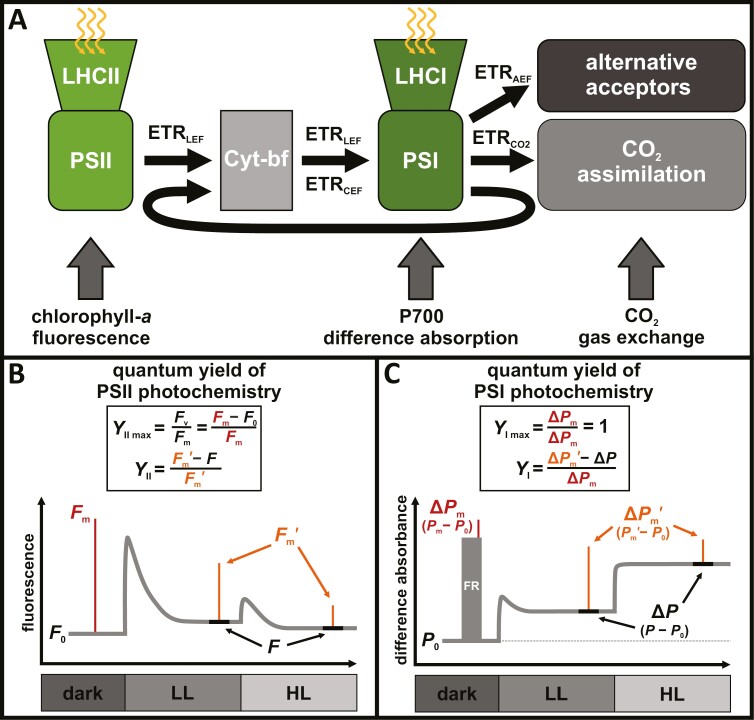
*In vivo* measurements of the electron transport chain components. (A) Simplified scheme showing the components of the photosynthetic electron transport chain facilitating linear electron flow and associated electron transport rates (ETR) for various electron pathways. All electron transport reactions are driven by charge separation in the reaction centers of photosystem I (PSI) and photosystem II (PSII), after light harvesting by their respective antenna systems (LHCI and LHCII). The major electron transport route is the linear electron flow (ETR_LEF_) from PSII via the cytochrome *b*_6_*f* (Cyt-*bf*) complex to PSI. ETR_LEF_ is partitioned into either ETR_CO2_, used for CO_2_ fixation in the Calvin–Benson–Bassham cycle, or alternative electron flow (ETR_AEF_), leading to reduction of alternative electron acceptors. Additionally, electrons can be shuttled from PSI back into the plastoquinone pool via cyclic electron flow (ETR_CEF_). At the leaf level, the functional state of the components of the electron transport chain can be probed by different *in vivo* measurements: for PSII via pulse-amplitude-modulated (PAM) chlorophyll *a* fluorescence, for PSI via P700 difference absorbance, and for CO_2_ fixation via CO_2_ gas exchange. These measurements provide information about the different photosynthetic partial reactions and, if combined, about the partitioning into different electron pathways. In particular, steady-state ETR_CEF_ is commonly estimated by the difference of electron transport rates through PSII and PSI, calculated from the quantum yields of PSII and PSI photochemistry. (B, C) Determination of quantum yields of PSII and PSI photochemistry relies on the application of short saturating pulses (SP, red and orange), which briefly change the apparent fluorescence/difference absorbance signal (gray), typically applied at different light intensities during a light curve experiments [e.g. from dark to low light (LL) and high light (HL)]. These SPs allow the determination of quantum yields of photochemistry, which are generally differentiated between maximal (*Y*_II max_ or *Y*_I max_) and effective yield of photochemistry (*Y*_II_ or *Y*_I_). *Y*_II max_ or *Y*_I max_ are associated with SP-induced (red) amplitude changes to the reference signal in darkness (*F*_m_ and *F*_0_ for PSII; Δ*P*_m_ for PSI, the latter assisted by pre-illumination with far-red (FR) light), which ensures the estimation of maximal yields of only ‘open’ reaction centers in absence of non-photochemical processes, like reversible non-photochemical quenching in PSII and non-photochemical processes associated with donor- and acceptor-side limitation in PSI. Similarly, *Y*_II_ or *Y*_I_ are associated with SP-induced (orange) amplitude changes to the steady-state signal in the light (*F*_m_ʹ and *F* for PSII; Δ*P*_m_ʹ and Δ*P* for PSI), which allows the estimation of the effective yields in the light, dependent on the ‘closure’ of reaction centers and non-photochemical processes. Despite these similarities, the direct comparison of the definitions of quantum yields of photochemistry in PSII (B) and in PSI (C) reveals that *Y*_II max_ (*F*_v_/*F*_m_) is variable, while the equivalent *Y*_I max_ is assumed to be constant. Ultimately, this leads to the neglecting of PSI photoinhibition and to inevitable distortions of the yield of photochemistry in PSI (and other PSI quantum yields) compared with PSII, because PSI quantum yields are expressed relative to maximal redox active fraction of PSI (Δ*P*_m_).

Investigating the partitioning of different photosynthetic electron transport pathways relies on *in vivo* measurements, generally including gas exchange measurements for assessing CO_2_ assimilation, as well as pulse-amplitude-modulated (PAM) chlorophyll *a* fluorescence and P700 difference absorbance measurements (Fig. 1A) to determine quantum yields of photochemistry and estimate electron transport rates (ETR) in PSII and PSI, respectively (Harbinson and Foyer, 1991; Laisk and Loreto, 1996; Joliot and Joliot, 2002; Laisk *et al.*, 2002; Morales *et al.*, 2018; Walker *et al.*, 2020; Yin *et al.*, 2021). Comparison of quantum yields and ETRs between PSII and PSI has been widely used to track their functional dynamics, including CEF (Harbinson *et al.*, 1989; Harbinson and Foyer, 1991). Nevertheless, this approach has several well-documented shortcomings (Fan *et al.*, 2016) that, as a first approximation, have been widely assumed to remain constant and include, for example, leaf absorption, excitation energy distribution between PSII and PSI, sampling depth within the leaf tissue, and contributions of plastocyanin and ferredoxin to the P700 difference absorbance signal. Although these assumptions deserve further attention, the focus of the present study is an independent factor that has remained much less documented—the photoinhibition of PSI. PSI photoinhibition can affect the estimation of PSI quantum yields from P700 measurements (Zivcak *et al.*, 2015; Kanazawa *et al.*, 2017; Lempiäinen *et al.*, 2022) and can be also very dynamic, which could interfere with our ability to disentangle the dynamics of PSII and PSI in boreal evergreen conifers on the seasonal scale. The approaches for estimating the quantum yields of PSII and PSI may appear very similar at first glance, as both are based on PAM techniques and saturating pulses, which quantifies amplitude changes between minimal (*F*_0_ or *P*_0_), steady-state (*F* or *P*) and maximal signal levels (*F*_m_, *F*_m_ʹ or *P*_m_, *P*_m_ʹ) (Genty *et al.*, 1989; Klughammer and Schreiber, 1994). However, the biophysical principles and assumptions underlying the interpretation of the signals are generally different for PSII and PSI. These differences can be readily identified by directly comparing the definitions of the photochemical yields of PSII (Fig. 1B) and PSI (Fig. 1C), since both are equivalent expressions of the maximal (*Y*_II max_ and *Y*_I max_) and effective activity of the photosystems (*Y*_II_ and *Y*_I_).

For PSII, these are derived from chlorophyll *a* fluorescence changes, which directly relate to the energy distribution within PSII (Butler, 1978; Genty *et al.*, 1989), while for PSI, they are derived from P700 difference absorbance measurements, which relate to the active PSI fraction (not the total PSI content) and redox states of the PSI reaction center and its acceptors (Klughammer and Schreiber, 1994, 2008). However, unlike for PSII, PSI yields are normalized to the maximal photo-oxidizable P700 pool (Δ*P*_m_), which we refer to as the maximal redox active fraction of PSI. Critically, the original definition of PSI quantum yields assumes a constant *Y*_I max_, while the equivalent expression for PSII (*Y*_II max_, typically referred to as *F*_v_/*F*_m_) is variable.

The assumption of a constant *Y*_I max_ inevitably leads to a definition of PSI quantum yields that does not account for PSI photoinhibition, which is rather surprising given that PSI photoinhibition is widely accepted to be congruent with a decrease of the maximal redox active PSI fraction or Δ*P*_m_ (e.g. Havaux and Davaud, 1994; Terashima *et al.*, 1994; Ivanov *et al.*, 1998; Sonoike, 1999; Kim *et al.*, 2001; Zhang and Scheller, 2004; Sejima *et al.*, 2014; Tikkanen and Grebe, 2018). Neglecting PSI photoinhibition can lead to distortion of PSI quantum yields (Zivcak *et al.*, 2015; Kanazawa *et al.*, 2017; Lempiäinen *et al.*, 2022), which has previously been described as a ‘funnel effect’ of PSI (Fan *et al.*, 2008). If not accounted for, this phenomenon could severely limit our ability to resolve the regulation of PSI and the relationship of PSI and PSII yields, in particular under condition where PSI photoinhibition cannot be excluded *a priori*, such as in overwintering boreal conifers.

The aim of this study was to elucidate the seasonal dynamics of PSI and PSII in boreal evergreen conifers. To this end, we followed the spring recovery of photosynthesis in Scots pine (*Pinus sylvestris*) and Norway spruce (*Picea abies*), two members of the *Pinaceae* family, growing in a boreal forest tree stand in Southern Finland from February to July 2017 by combining *in vivo* CO_2_ gas exchange, chlorophyll *a*, and P700 difference absorbance measurements in conjunction with semi-quantitative immunoblots of photosynthetic thylakoid proteins. Importantly, we introduced and applied a set of corrected PSI quantum yields that extended the analysis of P700 difference absorbance measurements and allowed the identification of seasonal PSI photoinhibition in boreal conifers.

## Materials and methods

### Plant material and study side

Three adult Scots pine (*Pinus sylvestris*, hereafter pine) and three Norway spruce (*Picea abies*, hereafter spruce) trees growing in SMEAR II (Station for Measuring Forest Ecosystem-Atmosphere Relations) in Hyytiälä, Southern Finland (61°51ʹN, 24°17ʹE, 180 m a.s.l.), were used for repeatedly collecting samples during the spring recovery of photosynthesis between February and July 2017 approximately every 2 weeks (total of 10 sampling points). At each sampling point, small shoots were cut from sun-exposed top canopy branches (16–25 m height) with the help of permanently installed scaffolding towers, and rapidly brought to the laboratory where they were recut under water within 10–15 min, awaiting further measurements. In parallel, small batches of mature needles were immediately sampled into cryotubes upon arrival in the laboratory and frozen in liquid nitrogen for later thylakoid isolations. For practical reasons, pine and spruce samples were measured during consecutive days [day of year (DOY) for pine and DOY+1 for spruce] at DOY 46, 67, 81, 95, 109, 124, 137, 151, 172, and 193. All sampled material corresponded to current-year needles (developed during summer of 2016).

### Thylakoid isolation and chlorophyll determination

Thylakoid isolations were performed under dim light at 10 °C with ice-cold reagents, as previously described (Grebe *et al.*, 2019). Chlorophyll concentrations of isolated thylakoids were determined in buffered 80% (v/v) acetone (Porra *et al.*, 1989).

### Gel electrophoresis and immunoblotting

Isolated thylakoids were solubilized in Laemmli buffer (Laemmli, 1970), loaded on an equal chlorophyll basis and separated by SDS-PAGE (6 M urea, 12% (w/v) acrylamide). For immunoblotting, proteins were transferred on polyvinylidene difluoride membrane (Millipore) and recognized by specific antibodies for PSI core (reaction center) protein PsaB (AS10695, Agrisera), PSII core (reaction center) protein D1 (DE-loop, gift from Prof. Roberto Barbato), PSII core (inner-antenna) protein CP47 (gift from Prof. Roberto Barbato), trimeric LHCII antenna protein LHCB2 (AS01003, Agrisera), cytochrome *b*_6_*f* complex protein PetA (AS08306, Agrisera), flavodiiron protein FLVA (custom antibody against *P. abies*, Agrisera), flavodiiron protein FLVB (custom antibody against *P. abies*, Agrisera), PSII accessory protein PSB27 (gift from Dr Lixin Zhang) and PSII accessory protein HCF244 (PHY0327, PhytoAB). For detection, horseradish peroxidase-linked secondary antibody (Agrisera) and Amersham ECL western blotting detection reagents (GE Healthcare) were used. Relative quantification of PsaB antibody signals was performed with ImageJ (Schneider *et al.*, 2012) from scanned immunoblots.

### CO_2_ gas exchange measurements

Photosynthetic CO_2_ gas exchange measurements were performed with a portable IRGA (GFS-3000, Walz) as described previously (Rajewicz *et al.*, 2023). Maximal CO_2_ assimilation rates (*A*_max_) were recorded under 1500 µmol photons m^−2^ s^−1^ photosynthetically active radiation (PAR) and 1500 ppm CO_2_ at 20 °C after stabilization.

### *In vivo* chlorophyll *a* fluorescence and P700 difference absorbance measurements

Simultaneous *in vivo* PAM chlorophyll *a* fluorescence (>700 nm) and P700 difference absorbance (Δ*I*_830–870nm_/*I*_830–870nm_) measurements (Dual-PAM-100, Walz) were performed on matts of detached needle leaves at room temperature after 1 h dark acclimation at 10 °C with custom-made needle adapters (Supplementary Fig. S1). Adapters were used to improve comparability of *in vivo* measurements by ensuring minimal gap size and parallel alignment of needle leaves. Fluorescence measurement light intensity was set to <1.0 µmol photons m^−2^ s^−1^ PAR and saturating pulse (SP) intensity was 8000 µmol photons m^−2^ s^−1^ PAR with a pulse width of 700 ms. Initial *F*_m_ and *F*_0_ were recorded directly after dark-acclimation with a SP, followed by Δ*P*_m_ determination with a SP after 10 s of far-red (FR) pre-illumination (130 μmol photons m^−2^ s^−1^, 720 nm). Samples were then subjected to a light curve protocol with 3 min intervals of increasing actinic light intensities (635 nm) in six steps of 25, 50, 100, 400, 800 and 1200 µmol photons m^−2^ s^−1^ PAR, each followed by a SP.

Fast kinetics of Δ*P*_m_ determinations were manually checked for anomalies (Supplementary Fig. S2A, B) and showed varying amounts of P700 reoxidation during SP illumination, consistent with activity of FLV proteins (Ilík *et al.*, 2017). Due to seasonal changes in the initial oxidation rate of P700 leading to delay in the maximal oxidation of P700 (Supplementary Fig. S2C, D), *P*_m_ was directly determined from the largest difference absorbance amplitude within the first 30 ms of the saturating pulse instead of default interpolation from the slope of the reduction phase (Klughammer and Schreiber, 2008), which prevented underestimation of Δ*P*_m_. In contrast, *P*_m_ʹ was determined according to the interpolation method with default delay time and width of 5 ms and 30 ms, respectively.

### Calculation of quantum yields and cyclic electron transport

PSII quantum yields were calculated according to Porcar-Castell (2011) where *Y*_II max_ (or *F*_v_/*F*_m_) is quantum yield of maximal PSII photochemistry [*Y*_II max_=1−(*F*_0_/*F*_m_)]; *Y*_II_ is quantum yield of effective PSII photochemistry [*Y*_II_=1−(*F*/*F*_m_ʹ)]; *Y*_NO_ is quantum yield of basal energy dissipation (*Y*_NO_=*F*/*F*_mR_); *Y*_NPQr_ is quantum yield of reversible non-photochemical quenching [*Y*_NPQr_=(*F*/*F*_m_ʹ)−(*F*/*F*_m_)]; and *Y*_NPQs_ is quantum yield of sustained non-photochemical quenching [Y_NPQs_=(*F*/*F*_m_)−(*F*/*F*_mR_)]. *F*_mR_ denotes the reference maximal fluorescence level in the absence of sustained and reversible non-photochemical quenching (NPQs and NPQr) and was interpolated from *F*_m_ measurements of summer samples (DOY 151–193) by assuming *Y*_II ref_ of 0.87, corresponding to the largest *Y*_II max_ observed in both species (Porcar-Castell, 2011). Classical PSI quantum yields were calculated according to Klughammer and Schreiber (2008), with quantum yield of non-photochemical dissipation due to PSI donor-side limitation (*Y*_ND_=Δ*P*/Δ*P*_m_), quantum yield of effective PSI photochemistry [*Y*_I_=(Δ*P*_m_ʹ−Δ*P*)/Δ*P*_m_], and quantum yield of non-photochemical dissipation due to PSI acceptor-side limitation [c*Y*_NA_=(Δ*P*_m_−Δ*P*_m_ʹ)/Δ*P*_m_]. Corrected PSI quantum yields were calculated as detailed in section ‘Derivation of corrected PSI quantum yields’ with corrected quantum yield of non-photochemical dissipation due to PSI donor-side limitation (c*Y*_ND_=ΔP/Δ*P*_mR_), corrected quantum yield of effective PSI photochemistry [c*Y*_I_=(Δ*P*_m_ʹ−ΔP)/Δ*P*_mR_], corrected quantum yield of non-photochemical dissipation due to PSI acceptor-side limitation [c*Y*_NA_=(Δ*P*_m_−Δ*P*_m_ʹ)/Δ*P*_mR_] and corrected quantum yield of non-photochemical energy dissipation due to PSI photoinhibition [c*Y*_PI_=(Δ*P*_mR_−Δ*P*_m_)/Δ*P*_mR_]. Δ*P*_mR_ denotes the reference maximal redox active PSI fraction ([P700]_R_^active^) and refers to the largest Δ*P*_m_ or largest maximal redox active PSI fraction [P700]^active^ per biological replicate and species observed during the sampling period (Supplementary Fig. S3). Cyclic electron flow was estimated from the difference in electron transport rates of PSI and PSII (ETR_CEF_=ETR_I_−ETR_II_). ETR_I_ and ETR_II_ were calculated as ETR_I_=*Y*_I_×*a*_I_×abs.×PAR and ETR_II_=*Y*_II_×*a*_II_×abs.×PAR, assuming equal distribution of excitation energy between photosystems (*a*_I_=*a*_II_=0.5) and constant leaf absorption (abs.=0.84).

### Statistical analysis

Statistically significant differences between sampling points were analysed by robust test of equality of means (Welch’s ANOVA) followed by a multiple comparisons post-hoc test (Games–Howell, *P*<0.05) for both species individually. Statistically significant differences between corrected and uncorrected PSI quantum yields were analysed by Student’s paired *t*-test (*P*<0.05). Regression analysis of *Y*_I_ or c*Y*_I_ and *Y*_II_ was evaluated by coefficient of determination (*R*^2^=1−[Σ(*y*_*i*−_*ŷ*_*i*_)^2^/Σ(*y*_*i*−_*ȳ*)^2^]), root mean square error (RMSE=√[Σ(*y*_*i*−_*ŷ*_*i*_)^2^/*n*]) and bias (bias=Σ(*y*_*i*−_*ŷ*_*i*_)/*n*) using all data points from light curve experiments (*n*=180). Regression analysis of PsaB antibody signal and *in vivo* estimations the of maximal redox active PSI fraction (Δ*P*_m_) were evaluated by coefficient of determination (*R*^2^) and root mean square error (RMSE) using all sampling points and biological replicates per species (*n*=30). Statistical significance of linear regression slopes was analysed by Student’s unpaired *t*-test (slope≠0, *P*<0.05). All statistics were performed with SPSS (v26.0; IBM Corp., Armonk, NY, USA).

### Derivation of corrected PSI quantum yields

In the classical definition (Klughammer and Schreiber, 1994, 2008), PSI quantum yields are based on the redox active PSI fraction ([P700]^active^), which comprises the active PSI centers but not the total PSI content. [P700]^active^ is estimated from the maximum amplitude of the difference absorbance signal of PSI (Δ*P*_m_) during a SP after far-red pre-illumination, which is specific for light-induced oxidation of P700 (Schreiber *et al.*, 1988). In the light-acclimated state, the individual PSI quantum yields each represent a relative subfraction of [P700]^active^ with different redox states of the PSI reaction center (P700) and its acceptors (A). These correspond to relative subfraction of Δ*P*_m_ defined by different absorbance levels estimated from a SP (Δ*P*_m_ʹ) or steady-state level (Δ*P*) during actinic illumination (for details, see Klughammer and Schreiber, 2008). It follows that the quantum yield of non-photochemical energy dissipation related to PSI donor-side limitation (*Y*_ND_) is associated to donor-side limited (closed) PSI centers with oxidized P700 and oxidized acceptors [P700^+^ A]:


$$\begin{array}{l} Y_{ND}=\frac{\left[P700^{+}\;\;\;A\right]\;\;\;}{{\left[P700\right]}^{active}}=\frac{\;\Delta P\;\;\;}{\;\Delta P_{m}}\;\;\; \end{array}$$ (1)

The quantum yield of effective PSI photochemistry (*Y*_I_) is associated to open PSI centers with reduced P700 and oxidized acceptors [P700 A]:


$$\begin{array}{l} Y_{I}=\frac{\left[P700\;\;\;A\right]\;\;\;}{{\left[P700\right]}^{active}}=\frac{\;\Delta P_{m}\prime -\;\Delta P}{\;\Delta P_{m}}\;\;\; \end{array}$$ (2)

The quantum yield of non-photochemical energy dissipation related to PSI acceptor-side limitation (*Y*_NA_) is associated to acceptor-side limited (closed) PSI centers with oxidized P700 and reduced acceptors [P700 A^−^]:


$$\begin{array}{l} Y_{NA}=\frac{\left[P700\;\;\;A^{-}\right]\;\;\;}{{\left[P700\right]}^{active}}=\frac{\;\Delta P_{m}-\;\Delta P_{m}\prime \;\;\;}{\;\Delta P_{m}}\;\;\; \end{array}$$ (3)

This leads to a strictly relative expression of the PSI quantum yields:


$$\begin{array}{l} Y_{ND}+Y_{I}+Y_{NA}=\frac{\;\Delta P\;\;\;}{\;\Delta P_{m}}+\frac{\;\Delta P_{m}\prime -\;\Delta P}{\;\Delta P_{m}}+\frac{\;\Delta P_{m}-\;\Delta P_{m}\prime \;\;\;}{\;\Delta P_{m}}=1 \end{array}$$ (4)

This is generally justified in non-stress conditions, because *Y*_I_ becomes the maximal PSI quantum yield of photochemistry (*Y*_I max_) equal to 1.0 in the absence of *Y*_ND_ and *Y*_NA_ (Δ*P*_m_ʹ=Δ*P*_m_ and Δ*P*=0):


$$\begin{array}{l} Y_{I\;max}=\frac{{\left[P700\right]}^{active}\;}{{\left[P700\right]}^{active}}=\frac{\;\Delta P_{m}\;}{\Delta P_{m}}=1\;(if,\;\Delta P_{m}\prime =\Delta P_{m}\;and\;\Delta P=0) \end{array}$$ (5)

This appears to be a reasonable approximation for the true maximal PSI quantum yield of photochemistry estimated to be 0.95–0.99 (Caffarri *et al.*, 2014).

**Fig. 2.**
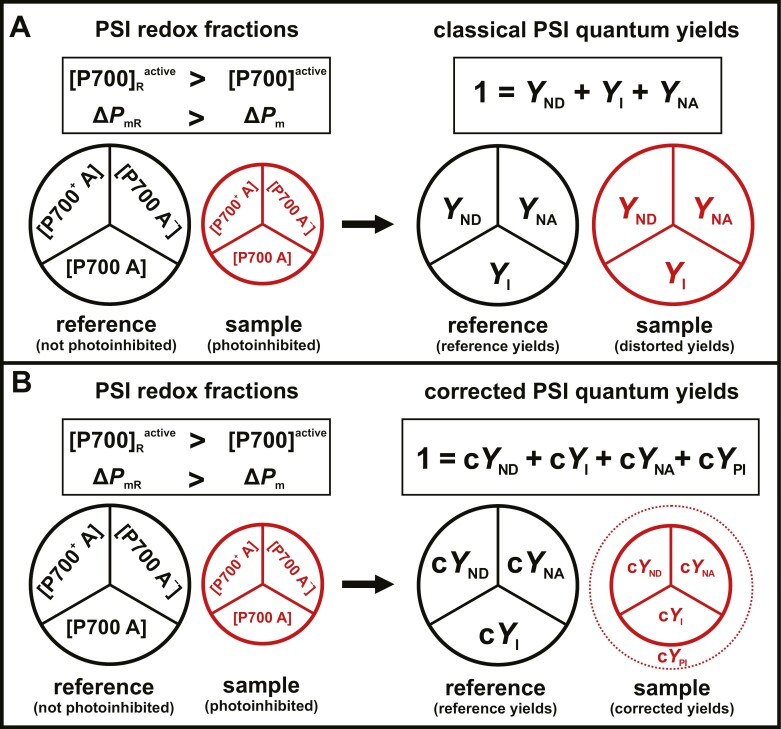
Relationship of photosystem I (PSI) redox fractions and PSI quantum yields during PSI photoinhibition. PSI quantum yields are based on the determination of the maximal redox active fraction of PSI ([P700]^active^) estimated as Δ*P*_m_, which is further divided into subfractions of different redox states of the PSI reaction center and its acceptors (left) corresponding to individual PSI quantum yields (right). Individual PSI redox fractions are differentiated by donor-side limited PSI centers with oxidized P700 and oxidized acceptors [P700^+^ A] corresponding to yield of non-photochemical energy dissipation related to PSI donor-side limitation (*Y*_ND_), open PSI centers with reduced P700 and oxidized acceptors [P700 A] corresponding to yield of effective PSI photochemistry (*Y*_I_), and acceptor-side limited (closed) PSI centers with oxidized P700 and reduced acceptors [P700 A^−^] corresponding to yield of non-photochemical energy dissipation related to PSI acceptor-side limitation (*Y*_NA_). However, when multiple P700 difference absorbance measurements with different [P700]^active^ or Δ*P*_m_ are compared [e.g. non-photoinhibited reference (black) and photoinhibited sample (red)], these definitions can lead to a discrepancy between the relationships of PSI redox fractions and their corresponding quantum yields. (A) Classical PSI quantum yields (Klughammer and Schreiber, 1994, 2008) do not consider differences in [P700]^active^ or Δ*P*_m_ between a non-photoinhibited reference (black) and photoinhibited sample (red). Ultimately, this leads to distortion of classical PSI quantum yields compared with their respective PSI redox fractions because the classical yields are expressed relative to [P700]^active^ or Δ*P*_m_. (B) Newly derived corrected PSI quantum yields accounted for differences in [P700]^active^ or Δ*P*_m_ between a non-photoinhibited reference (black) and photoinhibited sample (red). This is achieved by expressing the corrected PSI quantum yields relative to the maximal redox active PSI fraction of the reference ([P700]_R_^active^ or Δ*P*_mR_), which retains the expected relationship between the PSI redox fractions and their respective PSI quantum yields. Additionally, this gives rise to the quantum yield of non-photochemical dissipation due to PSI photoinhibition (c*Y*_PI_) accounting for the smaller maximal redox active PSI fraction relative to the reference.

However, while it seems plausible to assume that *Y*_I max_ would remain similar and close to the true maximal yield between replicate samples, Equations 4 and 5 intrinsically neglect the potential effect of PSI photoinhibition, leading to a decrease of [P700]^active^ or Δ*P*_m_, between different samples. Since Equation 5 effectively assumes a constant *Y*_I max_ between different samples, this paradoxically leads to an overestimation of PSI quantum yields during PSI photoinhibition, relative to their actual redox active fractions (Fig. 2A). Accordingly, we propose a correction of PSI quantum yields to account for the effect of photoinhibition on [P700]^active^ and Δ*P*_m_, thereby preserving the changes in the actual redox active fractions (Fig. 2B).

This is achieved by expressing the PSI quantum yields not relative to [P700]^active^ or Δ*P*_m_ of each individual sample, but instead, relative to a single reference sample with [P700]_R_^active^ or Δ*P*_mR_, which is assumed to be not affected by PSI photoinhibition ([P700]_R_^active^ ≥ [P700]^active^ or Δ*P*_mR_ ≥ Δ*P*_m_). Such a reference sample can be selected from a reference condition or, for measurements on the seasonal scale, be equivalent to the largest Δ*P*_m_ observed during the sampling period. In either case, the use of a single Δ*P*_mR_ makes corrected PSI quantum yields a simple extension of the classical PSI quantum yields, for which Equations 1–5 are assumed to be true only for the reference sample. In other words, while classical PSI quantum yields assume absence of PSI photoinhibition in all samples, corrected PSI quantum yields assume the absence of PSI photoinhibition only in a single reference sample. It follows that for corrected PSI quantum yields, the maximal quantum yield of photochemistry of the reference (*Y*_I ref_) can be expressed similar to Equation 5:


$$\begin{array}{l} Y_{I\;\;ref}=\frac{[P700{]}_{R}^{active}}{[P700{]}_{R}^{active}}=\frac{\;\Delta P_{mR}}{\;\Delta P_{mR}}=1 \end{array}$$ (6)

To account for PSI photoinhibition in any other sample, the corrected maximal quantum yield of PSI photochemistry (c*Y*_I max_) is expressed relative the reference sample, which reflects the ratio of [P700]^active^ to [P700]_R_^active^ or Δ*P*_m_ to Δ*P*_mR_:


$$\begin{array}{l} cY_{I\;max}=\frac{{\left[P700\right]}^{active}}{[P700{]}_{R}^{active}}=\frac{\;\Delta P_{m}}{\;\Delta P_{mR}} \end{array}$$ (7)

Since in the classical definition the sum of the individual PSI quantum yields (Equation 4) always equals *Y*_I max_ (Equation 5) in the absence of donor- or acceptor-side limitation, the complementary corrected PSI quantum yields can be derived by multiplying Equation 4 by the new c*Y*_I max_ (Equation 7) followed by transformation:


$$\begin{array}{lll} & \left(\frac{\Delta P}{\Delta P_{mR}}\right)+\left(\frac{\Delta P_{m}\prime -\Delta P}{\Delta P_{mR}}\right)+\left(\frac{\Delta P_{m}-\Delta P_{m}\prime }{\Delta P_{mR}}\right)+\left(\frac{\Delta P_{mR}-\;\Delta P_{m}}{\Delta P_{mR}}\right)\; & =cY_{ND}+cY_{I}+cY_{NA}+cY_{PI}=1 \end{array}$$ (8)

This corrected PSI quantum yield expression accounts for PSI photoinhibition by incorporating relative changes in [P700]^active^ to [P700]_R_^active^ or Δ*P*_m_ to Δ*P*_mR_. Importantly, the individual corrected yields of non-photochemical dissipation due to PSI donor-side limitation (c*Y*_ND_, Equation 9), quantum yield of effective PSI photochemistry (c*Y*_I_, Equation 10), and non-photochemical dissipation due to PSI acceptor-side limitation (c*Y*_NA_, Equation 11) retain their original definitions (Klughammer and Schreiber, 2008), but are simply expressed relative to [P700]_R_^active^ or Δ*P*_mR_:


$$\begin{array}{l} cY_{ND}=\frac{\left[P700^{+}\;\;\;A\right]\;\;\;}{[P700{]}_{R}^{active}}=\frac{\;\Delta P\;\;\;}{\;\Delta P_{mR}}\;\;\; \end{array}$$ (9)

$$\begin{array}{l} cY_{I}=\frac{\left[P700\;\;\;\;\;A\right]\;}{[P700{]}_{R}^{active}}=\frac{\;\Delta P_{m}\prime \;-\;\Delta P}{\;\Delta P_{mR}} \end{array}$$ (10)

$$\begin{array}{l} cY_{NA}=\frac{\left[P700\;\;\;\;\;A^{-}\right]\;\;\;}{[P700{]}_{R}^{active}}=\frac{\;\Delta P_{m}\;-\;\;\Delta P_{m}\prime \;}{\;\Delta P_{mR}} \end{array}$$ (11)

Additionally, a new PSI quantum yield c*Y*_PI_ is defined:


$$\begin{array}{l} cY_{PI}=\frac{[P700{]}_{R}^{active}\;\;\;-\;\;\;{\left[P700\right]}^{active}\;\;\;}{[P700{]}_{R}^{active}}=\frac{\;\Delta P_{mR}\;\;\;-\;\;\;\Delta P_{m}\;\;\;}{\;\Delta P_{mR}} \end{array}$$ (12)

It accounts for a non-photochemical energy dissipation due to PSI photoinhibition (e.g. decrease of maximal quantum yield of PSI photochemistry) by quantifying the relative decrease in the redox active PSI fractions of reference ([P700]_R_^active^) and sample ([P700]^active^).

Importantly, corrected PSI quantum yields (Equations 9–12) remain a simple extension of classical PSI quantum yields (Equations 1–3). This means that under non-photoinhibitory conditions ([P700]_R_^active^=[P700]^active^ or Δ*P*_m_=Δ*P*_mR_) both formulations are equivalent, because c*Y*_PI_ is zero and in turn classical and corrected yields become equal (c*Y*_ND_=c*Y*_ND_, c*Y*_I_=c*Y*_I_, c*Y*_NA_=c*Y*_NA_). Additionally, it should be noted that all (classical and corrected) PSI parameters have an ambiguous definition: (i) referring to energetic processes within PSI as quantum yields, and (ii) expressing changes to the redox active fractions of PSI. The strict interpretation as photochemical and non-photochemical processes remains problematic, as difference absorbance measurements cannot account for possible changes to the light harvesting efficiency or antenna size (Kanazawa *et al.*, 2017). Although we use the more common terminology of PSI quantum yields, we emphasize that the interpretations are based on changes of the redox active fraction and redox states of PSI.

## Results

**Fig. 3.**
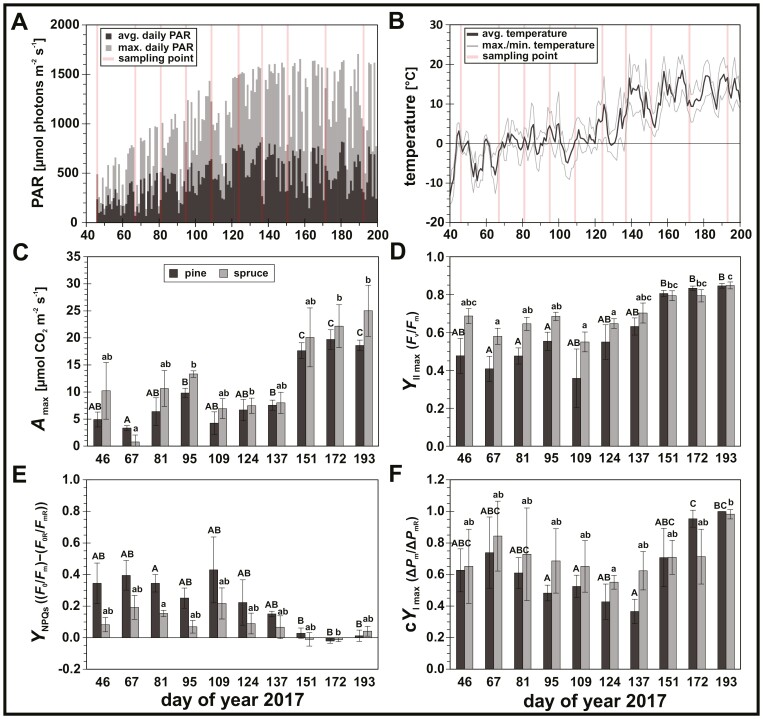
Seasonal changes in photosynthetically active radiation (PAR), daily temperature and functional photosynthetic capacities. (A) average daily PAR (400–700 nm), dark grey bars; maximal daily PAR (400–700 nm). (B) average daily temperature (thick grey line) and maximal and minimal average daily temperature (thin grey lines). Red lines mark sampling point as day of year (DOY) 2017 (DOY 46–193). (C–F) seasonal changes in photosynthetic *in vivo* parameters: (C) maximal CO_2_ assimilation rate (*A*_max_, 1500 ppm CO_2_, 1500 µmol photons m^−2^ s^−1^ PAR); (D) maximal quantum efficiency of photosystem II (PSII) photochemistry (*Y*_II max_ or *F*_v_/*F*_m_); (E) quantum yield of sustained non-photochemical quenching (*Y*_NPQs_); (F) maximal quantum efficiency of photosystem I (PSI) photochemistry (*Y*_I max_ or Δ*P*_m_/Δ*P*_mR_). Dark grey bars, pine (*Pinus sylvestris*); light grey bars, spruce (*Picea abies*). Letters represent statistically significant groups (uppercase for pine, lowercase for spruce), which were individually tested per species (Welch’s ANOVA, Games–Howell, *P*<0.05; error bars denote SD, *n*=3).

We followed the photosynthetic acclimation of pine and spruce trees growing in a forest in southern Finland from February to July 2017. The study period from February (DOY 46) to July (DOY 193) covered the seasonal increase in PAR (Fig. 3A) and temperature (Fig. 3B) typical for the northern boreal latitudes, with low average daily light intensities (<250 µmol photons m^−2^ s^−1^ PAR) and average daily temperatures below 5 °C at the beginning of the study period (winter, DOY 46–67), followed by a gradual increase in daily temperatures up to 10 °C and average daily light intensities from 250 to above 750 µmol photons m^−2^ s^−1^ PAR (spring, DOY 81–137), before reaching summer levels (summer, DOY 151–193). Interestingly, the study period included two cold spells immediately preceding DOY 67 and DOY 109, when average daily temperatures remained below 0 °C for several days and maximum daily light intensities were above 600 µmol photons m^−2^ s^−1^ PAR.

### Seasonal photosynthetic acclimation modulates functional capacity of CO_2_, PSII and PSI reactions

Photosynthetic CO_2_ assimilation rates, measured at 1500 µmol photons m^−2^ s^−1^ PAR and 1500 ppm CO_2_, were used as a proxy of maximum assimilatory rates (*A*_max_; Fig. 3C). *A*_max_ showed an overall increase from winter (DOY 46–67) towards summer (DOY 151–193) both in pine and spruce, reaching up to 19.7 ± 1.8 and 25.0 ± 4.7 µmol CO_2_ m^−2^ s^−1^, respectively. During spring (DOY 81–137), the assimilation capacity was more variable, but on average lower compared with summer, although only significant for pine. Similar to *A*_max_, the maximal quantum yield of PSII photochemistry (*Y*_II max_; Fig. 3D) recovered from winter (DOY 46–67) towards summer (DOY 151–193). During spring (DOY 81–137), recovery of the maximal PSII activity was interrupted by the induction of NPQs (*Y*_NPQs_; Fig. 3E), which accounted for 34 ± 6% and 15 ± 2% (DOY 81) of light energy losses due to sustained quenching processes in pine and spruce, respectively. *Y*_NPQs_ also transiently increased during the second cold spell (DOY 109) in both species when low temperatures occurred together with already higher irradiance levels. In terms of PSI photochemistry, and given that seasonal changes in the maximal redox active PSI fraction (Δ*P*_m_; Supplementary Fig. S3) justified the use of newly defined corrected PSI quantum yields (Equations 9–12), we identified a significant reduction of the maximal quantum yield of PSI photochemistry (c*Y*_I max_; Fig. 3F) down to 0.37 ± 0.08 (DOY 137) in pine and 0.55 ± 0.04 (DOY 124) in spruce, strongly suggesting PSI photoinhibition during late spring.

### Accounting for seasonal photoinhibition of PSI reveals functional balance between PSII and PSI photochemistry

**Fig. 4.**
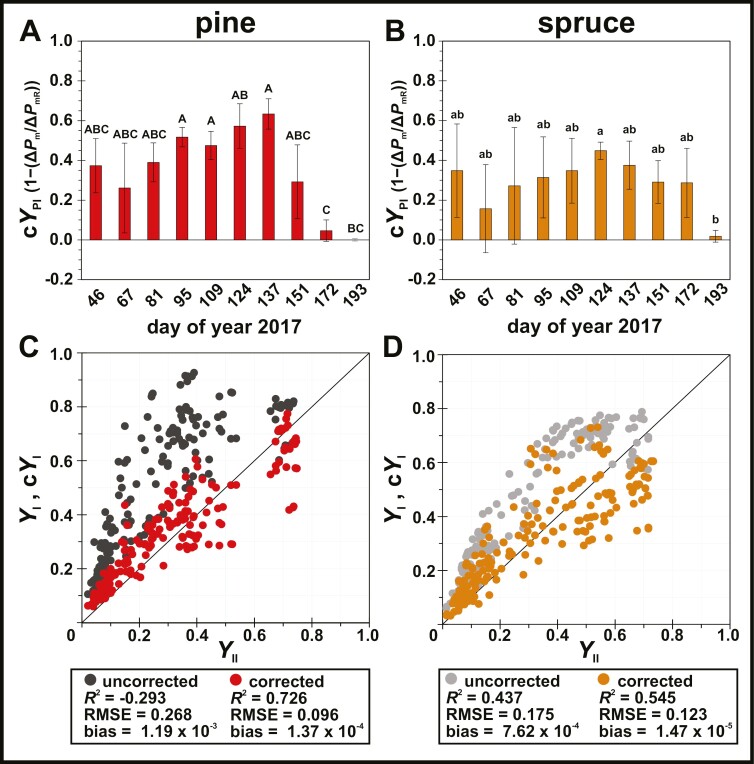
Influence of photosystem I (PSI) photoinhibition on the apparent functional dynamics of PSI and photosystem II (PSII). (A, B) non-photochemical dissipation due to PSI photoinhibition (c*Y*_PI_) accounting for the decrease of maximal redox active PSI fraction, in (A) pine (*Pinus sylvestris*, red), and (B) spruce (*Picea abies*, orange). Letters represent statistically significant groups (uppercase for pine, lowercase for spruce), which were individually tested per species (Welch’s ANOVA, Games–Howell, *P*<0.05; error bars denote SD, *n*=3). (C, D) Linear regression of quantum yields of effective photochemistry from PSI and PSII during actinic illumination, in (C) pine (*Pinus sylvestris*) with uncorrected *Y*_I_=*Y*_II_ (dark grey) and corrected c*Y*_I_=*Y*_II_ (red); and (D) spruce (*Picea abies*) with uncorrected *Y*_I_=*Y*_II_ (light grey) and corrected c*Y*_I_=*Y*_II_ (orange). Data points (*n*=180) from all sampling days [day of year (DOY) 46–193] and actinic light intensities (25, 50, 100, 400, 800, and 1200 µmol photons m^−2^ s^−1^ PAR) were used to calculate coefficient of determination (*R*^2^), root mean square error, and bias.

To account for PSI photoinhibition, we made use of corrected PSI quantum yields, which revealed major differences compared with classical PSI yields estimated from representative low light (LL, 100 µmol photons m^−2^ s^−1^ PAR) and high light (HL, 1200 µmol photons m^−2^ s^−1^ PAR) intensities of the light curve experiments. In both species, classical compared with corrected yields of effective PSI photochemistry (*Y*_I_ and c*Y*_I_; Supplementary Fig. S4A, B), donor-side limitation (*Y*_ND_ and c*Y*_ND_; Supplementary Fig. S4C, D) and acceptor-side limitation (*Y*_NA_ and c*Y*_NA_; Supplementary Fig. S4E, F) were significantly larger throughout the seasons. The overestimation was more dominant in pine compared with spruce and more frequent during spring (DOY 81–137), when PSI photoinhibition lead to a significant loss in maximal PSI activity (Fig. 3F). These seasonal dynamics of PSI photoinhibition could be quantified by the new PSI yield parameter c*Y*_PI_ (Fig. 4A, B) showing significant up-regulation during spring (DOY 81, 95, and 137 in pine; DOY 124 in spruce).

Accounting for seasonal PSI photoinhibition also affected the apparent relationship between PSI and PSII. The use of c*Y*_I_ instead of *Y*_I_ resulted in a better linear fit with *Y*_II_ from all light intensities and sampling points in pine (Fig. 4C) and spruce (Fig. 4D), consistent with the expected functional balance to support LEF. The use of c*Y*_I_ compared with *Y*_I_ resulted in an overall increase in the coefficient of determination (*R*^2^) and smaller residuals (RMSE), and reduced the positive bias by one order of magnitude in both species. The strongest differences were observed for spring sampling points (DOY 81–137), especially in pine, which was mostly affected by PSI photoinhibition (Supplementary Fig. S5). After correction for PSI photoinhibition, these results suggested that both pine and spruce maintained a more stable balance between PSII and PSI photochemistry than observed with the classical PSI quantum yield expressions. To gain a more comprehensive picture of the functional dynamics of PSII and PSI, we also analysed the seasonal patterns of PSII and PSI yields at LL and HL intensities in pine (Supplementary Fig. S6) and spruce (Supplementary Fig. S7). These highlighted that *Y*_NPQs_ and c*Y*_PI_ only changed on the seasonal scale, while regulation in response to illumination was dominated by *Y*_NPQr_ and c*Y*_ND_, leading to down-regulation of *Y*_II_ and c*Y*_I_ in HL compared with LL, respectively. In pine, the correction for PSI photoinhibition additionally shifted the apparent recovery phase of *Y*_I_ compared with c*Y*_I_ from spring (DOY 81–137) to summer (DOY 151–193), which more closely matched the recovery pattern of *Y*_II_ observed in LL and HL intensities.

**Fig. 5.**
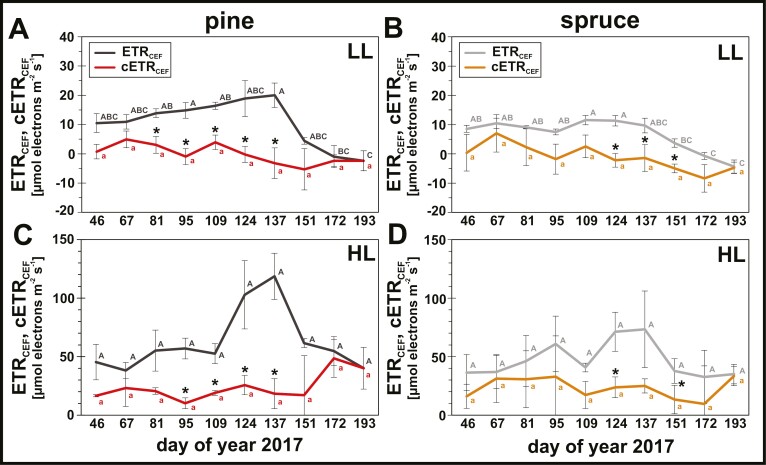
Influence of photosystem I (PSI) photoinhibition on seasonal estimations of steady-state cyclic electron flow (CEF). CEF was calculated as difference in electron transfer rate between PSI and photosystem II (PSII) (CEF=ETR_I_−ETR_II_) using either uncorrected *Y*_I_ (ETR_CEF_) or corrected c*Y*_I_ (cETR_CEF_). (A, B) ETR_CEF_ (dark/light grey) and cETR_CEF_ (red/orange) at 100 µmol photons m^−2^ s^−1^ PAR (low light, LL) of (A) pine (*Pinus sylvestris*) and (B) spruce *(Picea abies*). (C, D) ETR_CEF_ (dark/light grey) and cETR_CEF_ (red/orange) at 1200 µmol photons m^−2^ s^−1^ PAR (high light, HL) of (C) pine (*Pinus sylvestris*) and (D) spruce (*Picea abies*). *Statistically significant differences between ETR_CEF_ and cETR_CEF_ (paired *t*-test, *P*<0.05). Letters represent statistically significant groups (uppercase for ETR_CEF_, lowercase for cETR_CEF_), which were individually tested per parameter (Welch’s ANOVA, Games–Howell, *P*<0.05; error bars denote SD, *n*=3).

Furthermore, the correction of PSI quantum yields had a profound effect on the seasonal patterns of steady-state CEF, estimated from differences between the electron transport rates of PSI and PSII with *Y*_I_ (ETR_CEF_) or c*Y*_I_ (cETR_CEF_). In pine (Fig. 5A, C), cETR_CEF_ was strongly decreased compared with ETR_CEF_ during spring (DOY 81–137) in both LL and HL intensities. In spruce (Fig. 5B, D), the effects were less pronounced, but a significant effect was observed in late spring (DOY 124–137). Overall, cETR_CEF_ showed similar seasonal responses in both species, suggesting that after accounting for PSI photoinhibition neither species up-regulated steady-state CEF over the seasons.

### Seasonal variation in protein abundances related to photosynthetic light reactions

**Fig. 6.**
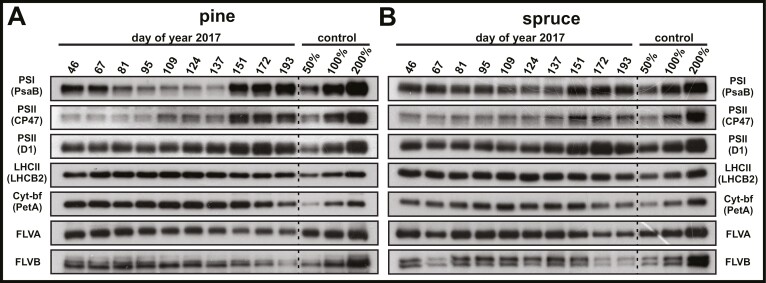
Seasonal changes in relative abundance of photosynthetic proteins. Representative immunoblots from thylakoid isolations of pine (*Pinus sylvestris*) (A) and spruce (*Picea abies*) (B) probed with PsaB (photosystem I), CP47 (photosystem II), D1 (photosystem II), LHCB2 (light harvesting complex II), PetA (cytochrome *b*_6_*f*), flavodiiron protein A (FLVA), and flavodiiron protein B (FLVB) antibodies. Samples were loaded on equal chlorophyll basis. Dilution series in percentage of thylakoid samples from DOY 193 (PsaB, CP47, D1, LHCB2, and PetA) and DOY 46 (FLVA and FLVB).

To further investigate the seasonal acclimation of the photosynthetic apparatus, we analysed changes in relative abundances of key photosynthetic proteins from pine (Fig. 6A) and spruce (Fig. 6B), separated from isolated thylakoids by SDS-PAGE (loaded on equal chlorophyll basis) and immunoblotted with protein specific antibodies. Immunoblots on an equal chlorophyll basis allowed us to investigate the seasonal acclimation of the photosynthetic apparatus on a per chloroplast level, which are better suited for comparisons of PSI and PSII dynamics in the context of photosynthetic energy balance than immunoblots on an equal protein basis (Walters, 2004).

The relative abundance of the PSI core protein PsaB showed a continuous decline in both species from winter (DOY 46–67) and throughout spring (DOY 81–137) until it rapidly increased during summer (DOY 151–193). Towards the end of spring (DOY 124–137), the PsaB abundance declined to well below 50% (approximately 20–30%) in pine and to around 50% in spruce in comparison with the respective summer control (DOY 193). Seasonal PsaB protein abundance also showed significant (*P*<0.05) linear correlations with maximal redox active PSI fractions (Δ*P*_m_). Without any assumptions about a quantitative connection between the two datasets (Supplementary Fig. S8A), the linear regression showed low overall fit (*R*^2^) and considerable variance (RMSE). However, since a quantitative connection between the maximal redox active PSI fraction and PSI content exists, because the difference absorbance signal of P700 is specific for PSI (Schreiber *et al.*, 1988), and each PSI center contains one P700 reaction center (Amunts *et al.*, 2010; Mazor *et al.*, 2015), the linear regression with intercept through the origin (Supplementary Fig. S8B) greatly improved the overall fit (*R*^2^) from 0.48 to 0.91 in pine and 0.25 to 0.81 in spruce with virtually no effect on the variance (RMSE).

Among the PSII proteins, the relative abundance of the PSII core protein CP47 remained low in both species during winter (DOY 46–67) and early spring (DOY 81–95) but increased towards the end of spring (DOY 109–137), particularly in pine, and substantially increased towards summer (DOY 151–193). A similar, but less pronounced, trend was also observed for the PSII core protein D1, which was expected because both proteins are PSII core subunits and therefore present in similar relative abundances in functional PSII complexes. Nevertheless, during winter (DOY 46–67) and early spring (DOY 81–95) an apparent higher abundance of D1 relative to CP47 was observed in comparison with their summer levels, suggesting a potentially active D1 re-synthesis via the PSII repair cycle in both species. The capacity for PSII repair, at least to some extent, during favorable winter (DOY 46–67) and spring (DOY 109–137) days in both species was supported by high relative levels of PSII accessory proteins, HCF244 and PSB27, involved in PSII repair and biogenesis (Supplementary Fig. S9A, B).

For LHCII, the immunoblots showed a relatively constant abundance of the trimeric LHCII antenna protein LHCB2 during winter (DOY 67) and spring (DOY 109–137), followed by a minor decline towards summer (DOY 151–193) in both pine and in spruce. Based on the apparent higher relative abundance of LHCII compared with PSII core (CP47) during winter (DOY 46–67) and spring (DOY 109–137), the immunoblots of both pine and spruce implied the presence of ‘extra’ LHCII, not tightly connected to PSII, during these periods. The cytochrome *b*_6_*f* complex (Cyt-*bf*) showed slightly different responses between the species based on immunoblots of PetA. In pine, relative Cyt-*bf* levels remained high throughout winter (DOY 46–67) and spring (DOY 81–137) followed by a decrease in summer (DOY 151–193). In spruce, relative Cyt-*bf* abundance increased from winter (DOY 46–67) to spring (DOY 81–137) and then decreased in summer (DOY 151–193). Additional immunoblots of thylakoid-associated flavodiiron proteins (FLVA and FLVB), involved in AEF, showed highest accumulations during winter (DOY 46–61) and spring (DOY 81–137). In pine, FLVA and FLVB gradually decreased already late spring (DOY 109–137), while in spruce the decrease was more rapid in summer (DOY 151–193).

**Fig. 7.**
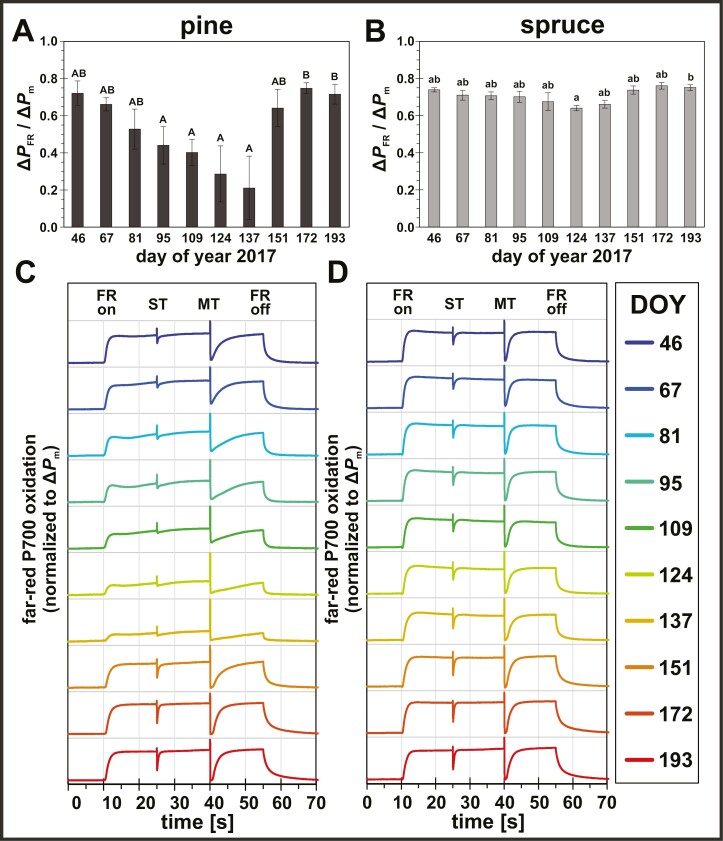
Seasonal changes in relative far-red (FR) induced P700 oxidation levels and kinetics. (A, B) FR-induced P700 oxidation level relative to Δ*P*_m_ in (A) pine (*Pinus sylvestris*) and (B) spruce (*Picea abies*). Letters represent statistically significant groups (uppercase for pine, lowercase for spruce), which were individually tested per species (Welch’s ANOVA, Games–Howell, *P*<0.05; error bars denote SD, *n*=3). (C, D) P700 oxidation kinetics (mean, *n*=3) during FR illumination, induced by single turnover (ST) and multiple turnover (MT) pulses, in (C) pine (*Pinus sylvestris*) and (D) spruce (*Picea abies*). Rainbow colors represent different sampling points from winter (blue) to summer (red). FR light intensity was 130 µmol photons m^−2^ s^−1^.

The opposing trends in PSI and PSII core proteins suggested seasonal adjustments of relative PSII:PSI stoichiometry. Particularly during late spring (DOY 109–137), the higher PSII (CP47) and lower PSI (PsaB) relative core protein abundances resulted in a high PSII:PSI ratio compared with winter (DOY 46–67), more prominent in pine compared with spruce. This was in line with independent functional PSI measurements of the far-red (FR) induced P700 oxidation. Although, FR illumination preferentially excites PSI, a small fraction of FR light is also absorbed by PSII. Consequently, the lower FR-induced P700 oxidation (Fig. 7A, B) and slower reoxidation kinetics (Fig. 7C, D) observed during late spring (DOY 109–137) indicated a larger electron flux from PSII towards PSI during FR illumination, which generally supported the observed higher PSII:PSI core protein stoichiometry during spring in pine and to a lesser extent also in spruce (Fig. 6).

## Discussion

### Spring photosynthesis of boreal conifers is characterized by PSI rather than PSII photoinhibition

The sampling period from February to July 2017 was characterized by gradual changes in light availability and temperature (Fig. 3A, B), during which both species showed typical recovery of *A*_max_ (Fig. 3C) and *Y*_II max_ (Fig. 3D) from winter to summer. During winter, *A*_max_ was not completely inhibited, which is consistent with previous reports of boreal evergreen conifers showing an opportunistic winter acclimation response by retaining minimal CO_2_ assimilation capacity to promptly utilize the warmer periods (Ensminger *et al.*, 2004; Kolari *et al.*, 2014). During spring, *A*_max_ and *Y*_II max_ were more variable in response to prevailing temperatures (Fig. 3A), in which cold spells can reverse and delay the photosynthetic recovery (Ensminger *et al.*, 2008; Wallin *et al.*, 2013). The lower *Y*_II max_ during winter and spring was consistent with the induction of *Y*_NPQs_ (Fig. 3E), known to be up-regulated in response to freezing temperatures (Porcar-Castell, 2011). Interestingly, we observed seasonal changes of Δ*P*_m_ (Supplementary Fig. S3), indicating a decrease in the maximal redox active PSI fraction, which justified the use of newly derived corrected PSI quantum yields (Equations 9–12). These allowed us to quantify c*Y*_I max_ (Fig. 3F), revealing a substantial decrease of the maximal PSI activity during spring in response to PSI photoinhibition in both species. These results also highlighted that in boreal evergreen conifers the preservation of maximal PSI activity during winter (Ivanov *et al.*, 2001; Öquist and Huner, 2003) is not necessarily maintained throughout the seasons.

During spring, the decline of c*Y*_I max_ (Fig. 3F) was in-line with the continuous decrease of the relative PSI core protein abundance in pine and spruce (PsaB, Fig. 6A, B) and generally supported by significant linear regressions of *in vivo* and immunoblot data (Supplementary Fig. S8), despite not being obtained from the same needle samples, which likely contributed to the large variance. The concomitant decline of both c*Y*_I max_ and PSI core protein abundance strongly suggests seasonal PSI photoinhibition in both pine and spruce, which, in the absence of an efficient PSI repair cycle (Scheller and Haldrup, 2005), is likely caused by photodamage of iron–sulfur clusters within PSI (Sonoike *et al.*, 1995; Tiwari *et al.*, 2016), followed by degradation of total PSI complexes dependent on ambient temperatures, as typically observed in angiosperms (Zhang and Scheller, 2004). The decline of PSI core proteins was contrasted by a relative increase of PSII core proteins (D1 and CP47; Fig. 6A, B) from winter throughout spring. Although PSII core protein content generally followed the recovery of *Y*_II max_ in pine and spruce (Fig. 3D), it remains difficult to directly connect seasonal PSII protein content to PSII photoinhibition, unlike for PSI. This is because (i) PSII photoinhibition is known to only accumulate when photodamage to the D1 protein exceeds the capacity of the efficient PSII repair cycle (Aro *et al.*, 1993), and (ii) both NPQs and PSII photoinhibition lead to seasonal decrease of *Y*_II max_, especially in conifers (Porcar-Castell, 2011; Grebe *et al.*, 2020).

It is therefore conceivable that, despite the up-regulation of NPQs (Fig. 3E), pine and spruce potentially suffered from photodamage over the whole season but did not accumulate large amounts of PSII photoinhibition due to a high capacity for PSII repair. This is supported by the apparent higher protein abundance of D1 compared with CP47 in both species during winter and spring compared with summer (Fig. 6A, B), which suggests increased D1 re-synthesis as part of the PSII repair cycle (Järvi *et al.*, 2015). In both pine and spruce, maintenance of D1 protein synthesis is additionally supported by a higher relative abundance of PSII accessory proteins during winter and spring (Supplementary Fig. S9A, B), with HCF244 functioning in D1 translation (Link *et al.*, 2012; Li *et al.*, 2019) and PSB27 being important for protection of PSII donor side prior to CP47 re-assembly (Chen *et al.*, 2006; Hou *et al.*, 2015; Zabret *et al.*, 2021).

### Boreal evergreen conifers maintain functional balance between photosystems despite changes to PSII:PSI stoichiometry

Lower PSI and PSII core protein contents during winter and spring compared with summer (Fig. 6A, B) generally agreed with previous studies following the spring recovery of boreal evergreens on an equal protein basis (Ensminger *et al.*, 2004; Verhoeven *et al.*, 2009; Verhoeven and Kornkven, 2023), albeit a direct comparison remains difficult due to differences in normalizations and seasonal light and temperature patterns between studies.

Our results here suggest large changes of the PSII:PSI stoichiometry compared with summer, ranging from less PSII relative to PSI core complexes during winter towards more PSII relative to PSI core complexes towards the end of spring, particularly in pine (Fig. 6A) compared with spruce (Fig. 6B). These PSII:PSI stoichiometry changes were supported by FR-induced P700 oxidation measurements, which are sensitive to the relative differences of functional PSII and PSI fractions (Losciale *et al.*, 2008). Consistent with a higher relative PSII to PSI content during spring, lower levels of P700 oxidation during FR illumination (Fig. 7A, B) and slower P700 reoxidation kinetics (Fig. 7C, D) indicated a larger electron flux from PSII towards PSI during spring, especially in pine. Changes in the PSII:PSI stoichiometry in both species were mainly driven by PSI, which seems to be a common mechanism in photosynthetic organisms (Murakami *et al.*, 1997a, b; Tullberg *et al.*, 2000), suggesting that PSI photoinhibition might be part of the seasonal acclimation strategy in boreal evergreen conifers.

It is important to note, that apparent changes in stoichiometries of PSII and PSI core complexes did not lead to large changes in chlorophyll content and leaf absorption, as shown in a previous analysis of the same needle samples and time points (Rajewicz *et al.*, 2023). This is in line with the notion that PSII:PSI stoichiometry changes are not necessarily detrimental for photosynthesis (Chow *et al.*, 1990). In our study, this is emphasized by the preservation of more balanced PSII and PSI effective yields of photochemistry throughout the seasons (Fig. 4C, D; Supplementary Fig. S5), which, however, was only apparent after correction for PSI photoinhibition (Fig. 4A, B; Supplementary Fig. S4). This apparent balance between photochemistry of PSII and PSI suggests that changes in stoichiometry of PSII and PSI core complexes in pine (Fig. 6A) and spruce (Fig. 6B) did not necessarily affect the excitation distribution between photosystems required for efficient LEF (Johnson and Wientjes, 2020). On the seasonal scale, the excitation distribution between photosystems is determined by their relative absorption cross-sections, which is not only dependent on the number of photosystems (core complexes) but also on the number of energetically connected antenna complexes per photosystem (antenna size). Therefore, it seems plausible that antenna size adjustments could compensate for altered relative antenna cross-sections caused by PSII:PSI stoichiometry changes of core complexes and thereby help to maintain a balanced excitation distribution over the seasons.

Since the amount of LHCI antenna proteins per PSI remains stable during long-term light acclimation (Schöttler and Tóth, 2014), the antenna size adjustments of both photosystems could be facilitated by an ‘extra’ LHCII pool not tightly associated with PSII. In angiosperms, the ‘extra’ LHCII pool is suggested to serve as a shared antenna for both photosystems (Wientjes *et al.*, 2013; Grieco *et al.*, 2015). Both pine and spruce showed higher relative LHCII (LHCB2) per PSII (CP47) content during winter and spring (Fig. 6A, B), which supports the presence of a larger ‘extra’ LHCII pool along with the observed changes in the PSII:PSI core stoichiometry. Seasonal antenna adjustments via an ‘extra’ LHCII pool do not necessarily rely on phosphorylated LHCII (like state transitions; Rochaix, 2014) but can be similarly facilitated by non-phosphorylated LHCII acting as an efficient antenna for PSI at different light conditions, as shown in angiosperms (Benson *et al.*, 2015; Bressan *et al.*, 2018; Bos *et al.*, 2019; Schiphorst *et al.*, 2022). Specifically for pine and spruce, their capacity to form very large PSII–LHCII supercomplexes (Kouřil *et al.*, 2020) might convey an overall greater flexibility to adjust their PSII antenna size and the ‘extra’ LHCII pool compared with other species to maintain a balanced excitation distribution over the season.

### Roles of alternative and cyclic electron flow during seasonal photosynthetic acclimation

AEF consists of different pathways, which divert electrons from LEF to alterative acceptors other than CO_2_. The majority of AEF pathways are oxygen dependent and are suggested to function as efficient electron sinks during excess light conditions (Alric and Johnson, 2017), because unlike CO_2_, the O_2_ concentration from the thylakoid level to the leaf level is in equilibrium with the environment (Ligeza *et al.*, 1997, 1998). In conifers, different AEF pathways like photorespiration (Busch, 2020), the Mehler reaction (Asada, 1999), plastid terminal oxidase (PTOX; Nawrocki *et al.*, 2015), and FLV proteins (Alboresi *et al.*, 2019a) have been suggested to protect the photosynthetic apparatus during winter and spring, although their differentiation *in vivo* still remains challenging (Savitch *et al.*, 2010; Bag *et al.*, 2023). Photorespiration is considered a major AEF pathway in C_3_ plants, due to its connection to the malate valve (Dao *et al.*, 2022), but is generally limited by low temperatures because the specificity of Rubisco for CO_2_ compared with O_2_ increases (Galmés *et al.*, 2016), and low temperatures also restrict the enzymatic reactions of the Calvin–Benson–Bassham cycle tightly in relation to photorespiration (Öquist and Huner, 2003; Chang *et al.*, 2021). PTOX and the Mehler reaction have an overall low electron flow capacity and their reactions also produce reactive oxygen species (Ort and Baker, 2002; Nawrocki *et al.*, 2015), suggesting that they play a stronger role in stress signaling (Li and Kim, 2022). This is in stark contrast to FLVs, which are not connected to reactive oxygen species production (Helman *et al.*, 2003; Allahverdiyeva *et al.*, 2011) and act as efficient electron acceptors protecting PSI (Ilík *et al.*, 2017).

While we do not exclude the possibility that other oxygen-dependent AEF pathways play a role during seasonal acclimation of conifers, the higher relative abundance of thylakoid-associated FLV proteins in winter and spring (Fig. 6A, B), when maximal CO_2_ assimilation capacities were overall low (Fig. 3C), suggests an elevated capacity for FLV-mediated AEF in both pine and spruce. The delayed decline of thylakoid-associated FLV abundances in spruce during spring potentially contributed to less severe seasonal PSI photoinhibition compared with pine (Fig. 3F), which generally agrees with a greater propensity for AEF in spruce compared with pine, previously found during artificial acclimation to elevated CO_2_ and temperature (Kurepin *et al.*, 2018).

Similar to AEF, also CEF around PSI is considered to be important to maintain photosynthesis under environmental stress conditions by balancing the ATP/NAPDH ratio and protecting both photosystems from photodamage (Yamori and Shikanai, 2016). In conifers, CEF has been suggested to be up-regulated during winter and spring (Ivanov *et al.*, 2001; Fréchette *et al.*, 2015; Yang *et al.*, 2020). While our estimation of steady-state CEF using classical PSI quantum yields (ETR_CEF_) supported these results, accounting for PSI photoinhibition with corrected PSI quantum yields (cETR_CEF_) showed no seasonal up-regulation of CEF in pine (Fig. 5A, C) and spruce (Fig. 5B, D).

Similar to previous conifer studies (Fréchette *et al.*, 2015; Yang *et al.*, 2020), our estimations of steady-state CEF assume equal excitation distribution between photosystems (*a*_1_=*a*_2_=0.5) and constant leaf absorption (*A*=0.84), which can lead to erroneous estimations of steady-state CEF (Fan *et al.*, 2016). However, since both these assumptions are independent from assumptions related to the calculations PSI quantum yields, both ETR_CEF_ and cETR_CEF_ are similarly affected. Although the absolute rates ETR_CEF_ and cETR_CEF_ are likely still error prone, the clear differences of CEF with or without correction for PSI photoinhibition advocate for a significant influence of seasonal PSI photoinhibition on the estimation of steady-state CEF in pine and spruce (Fig. 5), independently from other common error sources (Fan *et al.*, 2016).

Aside from the seasonal acclimation in conifers, it should be pointed out that PSI photoinhibition in plants is likely to take place under environmental stress conditions that have been widely associated with up-regulation of steady-state CEF. These include low temperatures (Sonoike, 1999; Huang *et al.*, 2011), low CO_2_ (Harbinson and Foyer, 1991; Miyake *et al.*, 2005), drought (Golding and Johnson, 2003), and high irradiance (Clarke and Johnson, 2001; Miyake *et al.*, 2004; Kou *et al.*, 2013; Huang *et al.*, 2015), all of which are likely to cause PSI acceptor side limitation ultimately leading to PSI photoinhibition (Sonoike, 2010; Lima-Melo *et al.*, 2021). Although these studies serve as the foundation for the physiological relevance of steady-state CEF during environmental stress conditions (Yamori and Shikanai, 2016), they do not consider the possible artificial inflation of PSI quantum yields during PSI photoinhibition (Fig. 2; Supplementary Fig. S3). Our results from boreal evergreen conifers suggest a need to revisit the physiological relationship between steady-state CEF and PSI photoinhibition.

### Critical assessment of proposed PSI quantum yield corrections

**Fig. 8.**
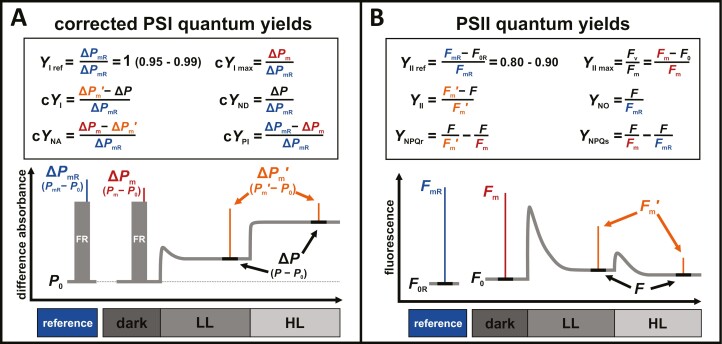
Formal analogies between corrected photosystem I (PSI) and photosystem II (PSII) quantum yields. Color coded parameter definitions (top) and schematic of experimental procedure (bottom) of PSI quantum yields (*Y*_I__ref_, c*Y*_I max_, c*Y*_I_, c*Y*_ND_, c*Y*_NA_, c*Y*_PI_) (A) and PSII quantum yields (*Y*_II ref_, *Y*_II max_, *Y*_II_, *Y*_NO_, *Y*_NPQr_, *Y*_NPQs_) (B) highlight formal analogies. Reference measurements (blue) of difference absorbance (Δ*P*_mR_) or fluorescence (*F*_mR_, *F*_0R_) refer to samples without PSI photoinhibition or sustained non-photochemical quenching (NPQs) and PSII photoinhibition, respectively.

We derived corrected PSI quantum yields (c*Y*_I_, c*Y*_ND_, c*Y*_NA_, c*Y*_PI_; see Equations 9–12) to account for variations in the maximal redox active PSI fraction (Δ*P*_m_) caused by PSI photoinhibition (Supplementary Fig. S3), which are not considered in the classical definition (Klughammer and Schreiber, 1994, 2008). Since PSI photoinhibition can be present under many environmental conditions (Lima-Melo *et al.*, 2021), our correction is necessary to avoid distortion of PSI yields (Zivcak *et al.*, 2015; Kanazawa *et al.*, 2017; Lempiäinen *et al.*, 2022) relative to their respective PSI redox fractions (Fig. 2; Supplementary Fig. S5). To achieve this, corrected yields are defined relative to a reference maximal redox active PSI fraction (Δ*P*_mR_), which ensures that the absence of PSI photoinhibition is only assumed for the reference instead of all samples during seasonal comparisons. Additionally, the definition of corrected PSI quantum yields (Fig. 8A) is more similar to that of PSII quantum yields (Fig. 8B), which also include a reference (Porcar-Castell, 2011). In both cases, the reference provides an approximation of the true maximal quantum yield of each photosystem (*Y*_I ref_=0.95–0.99≈1.0, *Y*_II ref_=0.80–0.90; Caffarri *et al.*, 2014), which facilitates the quantitative comparisons of functional PSI or PSII dynamics (Fig. 4C, D; Supplementary Fig. S5). In practice, this argumentation is similar to the introduction of classical PSI quantum yields with *Y*_NA_ (Klughammer and Schreiber, 1994, 2008), succeeding the original yield estimations of PSI photochemistry solely based on reduced P700 (Weis *et al.*, 1987; Harbinson *et al.*, 1989), which also leads to closer expected relationship of *Y*_I_ and *Y*_II_ (Klughammer and Schreiber, 1994).

The application of corrected PSI quantum yields relies on the quantitative comparison of P700 difference absorbance measurements (Δ*I*_830–870_/*I*_830–870_), in particular of Δ*P*_m_. For reliable comparisons of needle leaf measurements, it is important to minimize gaps between needles, which otherwise lead to overestimations. In this study, this is achieved by measurements of needle matts with custom adapters (Supplementary Fig. S1). Small needle gaps contributing to the observed variation Δ*P*_m_ levels between biological replicates (Supplementary Fig. S3) cannot be fully excluded and might be amplified in spruce compared with pine samples due to the use of a smaller measuring window. Additionally, the observed variations could also be of biological origin, reflecting different irradiance profiles between biological replicates and species caused by individual differences in shoot structures (Niinemets, 2007). Regardless, potential impacts of species differences and light-environment specific differences in leaf morphology and shoot structures were minimized by using a separate Δ*P*_mR_ value per species and biological replicate. Δ*P*_m_ comparisions can be further affected by changes in the structural absorption properties on the seasonal scale, including changes in leaf thickness (detour effects) and chloroplast movements (sieve effects). In this study, both influences have been minimized by restricting measurements to mature leaves, with only limited capacity to reacclimate their photosynthetic tissue (Niinemets, 2007), and the use of red actinic light, avoiding blue light-induced chloroplast movements (Jarillo *et al.*, 2001; Kagawa *et al.*, 2001; Sakai *et al.*, 2001). Additionally, Δ*P*_m_ estimations are affected by contributions of plastocyanin (PC) and ferredoxin to the P700 difference absorbance signal (Schansker *et al.*, 2003; Klughammer and Schreiber, 2016). In particular, the effects of PC content on the amplitude of Δ*P*_m_ might be non-negligible, despite its significant reduction by the dual-wavelength acquisition of the Dual-PAM-100 instrument (Klughammer and Schreiber, 1998; Kirchhoff *et al.*, 2004). Although we did not quantify the seasonal changes of PC protein contents in pine and spruce, previous work in *Pinus banksiana* suggests that PC does not decline as strongly as PSI protein contents under PSI photoinhibitory conditions (Busch *et al.*, 2008). Transferred to our study, residual PC protein content might therefore likely cause an overestimation of the total amplitude of Δ*P*_m_, which could explain the discrepancy between the observed 70–80% loss of PSI core proteins (Fig. 6A, B) leading to only 50–60% reduction of the maximal active PSI fraction (Δ*P*_m_; Supplementary Fig. S3) and maximal quantum yield of PSI photochemistry (c*Y*_I max_; Fig. 3F). Consequently, a deconvolution approach to distinguish between P700, PC, and Fd contributions (Klughammer and Schreiber, 2016; Schreiber and Klughammer, 2016) could improve the precision of Δ*P*_m_ determinations, albeit such an approach still needs more experimental controls (Sétif *et al.*, 2019, 2020), especially in non-model species. Nevertheless, such a deconvolution approach would still require a systemic correction of PSI quantum yields, as recently shown (Lempiäinen *et al.*, 2022), because the influence of PSI photoinhibition on the maximal redox-active PSI fraction is not considered.

Furthermore, it should be emphasized that the proposed correction still retains the ambiguous nature of the definition of classical PSI quantum yields (Kanazawa *et al.*, 2017), which is especially relevant for new parameter c*Y*_PI_. Since c*Y*_PI_ quantifies PSI photoinhibition as a decrease of the maximal redox active PSI fraction (Fig. 4A, B), its strict interpretation as a quantum yield could lead to the assumption of non-photochemical energy dissipation processes facilitated by a damaged PSI fraction, e.g. via charge recombinations (Matsuoka *et al.*, 2016; Milanovsky *et al.*, 2019). This creates a conundrum, in that the damaged PSI fraction promoting the non-photochemical processes might not be present in long-term experiments, as damaged PSI core proteins eventually become degraded (Zhang and Scheller, 2004). To avoid this problem, we strongly emphasize that P700 difference absorbance measurements should be interpreted strictly on the basis of relative changes to PSI fractions (Kanazawa *et al.*, 2017). Nevertheless, c*Y*_PI_ is still a useful parameter, when directly paired with c*Y*_I_, c*Y*_ND_, and c*Y*_NA_. Since c*Y*_PI_ effectively captures the decrease in c*Y*_I max_ caused by PSI photoinhibition, it readily translates variations of the maximal redox active PSI fraction to the analysis during actinic illumination (Supplementary Figs S6, S7).

### Concluding remarks

The photosynthetic recovery during spring in two boreal evergreen conifers, pine and spruce, demonstrated dynamics of CO_2_ assimilation capacity, activities of PSII and PSI, and thylakoid protein abundances. This prompted us to re-evaluate the PSI quantum yields, taking into consideration the seasonal influence of PSI photoinhibition, which has so far not been widely considered. Introducing and applying corrected PSI quantum yields allowed us to analyse the functional seasonal dynamics of PSII and PSI, free of distortions caused by PSI photoinhibition. Our results show that despite large PSII:PSI core protein stoichiometry changes, quantum yields of PSII and PSI photochemistry in pine and spruce remain much more in balance throughout the seasons than anticipated. With respect to photosynthetic electron transport, these results suggest a balanced operation of LEF between both photosystems throughout the seasons and do not support previous observations of seasonal up-regulation of CEF, probably caused by neglecting PSI photoinhibition.

Taken together, our results emphasize that the photosynthetic acclimation of light reactions in boreal evergreen conifers is not only governed by seasonal dynamics of PSII but also of PSI, which needs to be further elucidated. In this respect, our proposed correction of PSI yields expands the methodological toolkit to investigate PSI dynamics under PSI photoinhibitory conditions, also beyond the scope of boreal evergreen conifers.

## Supplementary data

The following supplementary data are available at *JXB* online.

Fig. S1. Technical details of custom-made needle adapters.

Fig. S2. Seasonal changes in fast-kinetics of maximal P700 oxidation during saturating pulse (Δ*P*_m_ determination).

Fig. S3. Seasonal change in maximal redox active PSI fraction (Δ*P*_m_) per biological replicate.

Fig. S4. Overestimation of uncorrected compared with corrected PSI quantum yields.

Fig. S5. Seasonal effect of PSI photoinhibition on the functional dynamics of PSI and PSII.

Fig. S6. Seasonal patterns of PSII and PSI quantum yields in pine (*Pinus sylvestris*).

Fig. S7. Seasonal patterns of PSII and PSI quantum yields in spruce (*Picea abies*).

Fig. S8. Linear regression of relative PSI abundance from thylakoid isolations (PsaB) and maximal redox active PSI fraction (Δ*P*_m_) from needle leaves *in vivo*.

Fig. S9. Seasonal changes in relative abundance of PSII accessory proteins.

erae145_suppl_Supplementary_Figures_S1-S9

## Data Availability

All data supporting the findings of this study are available within the paper and within its supplementary data published online. Any data not shown are available from the corresponding author, Eva-Mari Aro, upon request.
