# Supplementary material for: Accounting for photosystem I photoinhibition sheds new light on seasonal acclimation strategies of boreal conifers
Source: J Exp Bot. 2024 Apr 4;75(13):3973–92. doi: 10.1093/jxb/erae145 (PMC11233416; doi:10.1093/jxb/erae145)
Supplement: erae145_suppl_Supplementary_Figures_S1-S9 [file erae145_suppl_supplementary_figures_s1-s9.pdf]

Journal of Experimental Botany Supplementary Data

Accounting for photoinhibition of photosystem I elucidates the seasonal acclimation strategies of boreal conifers

Steffen Grebe[1,2], Albert Porcar-Castell[2], Anu Riikonen[2], Virpi Paakkarinen[1], Eva-Mari Aro[1]

[1]Molecular Plant Biology, Department of Life Technologies, University of Turku, 20014 Turku, Finland; [2]Optics of Photosynthesis Laboratory, Viikki Plant Science Center, Institute for Atmospheric and Earth System Research/Forest Sciences, University of Helsinki, 00014 Helsinki, Finland

The following Supplementary Data is available for this article:

Fig. S1 Technical details of custom-made needle adapters.
Fig. S2 Seasonal changes in fast-kinetics of maximal P700 oxidation during saturating pulse (ΔP_m determination).
Fig. S3 Seasonal change in maximal redox active PSI fraction (ΔP_m) per biological replicate.
Fig. S4 Overestimation of uncorrected compared to corrected PSI quantum yields.
Fig. S5 Seasonal effect of PSI photoinhibition on the functional dynamics of PSI and PSII.
Fig. S6 Seasonal patterns of PSII and PSI quantum yields in pine (*Pinus sylvestris*).
Fig. S7 Seasonal patterns of PSII and PSI quantum yields in spruce (*Picea abies*).
Fig. S8 Linear regression of relative PSI abundance from thylakoid isolations (PsaB) and maximal redox active PSI fraction (ΔP_m) from needle leaves *in-vivo*.
Fig. S9 Seasonal changes in relative abundance of PSII accessory proteins.

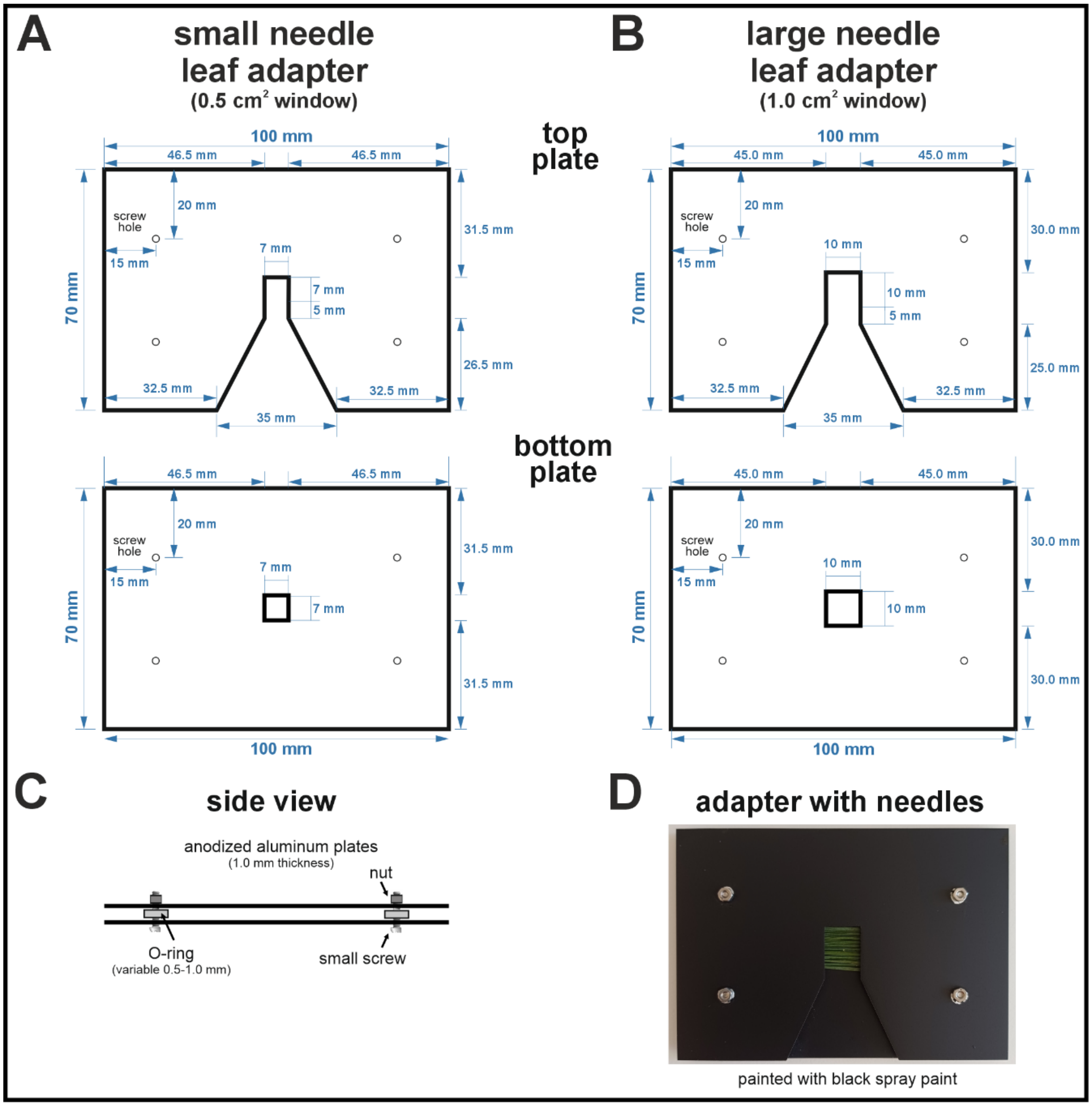

Fig. S1 Technical details of custom-made needle adapters. Needle leaf adapter used for *in-vivo* fluorescence and absorbance measurements with V-shape opening to allow rapid insertion and alignment of needles. **A**) technical drawing of top and bottom plate of small needle leaf adapter with 0.5 cm^2 measuring area used for spruce (*Picea abies*). **B**) technical drawing of top and bottom plate of large needle leaf adapter with 1.0 cm^2 measuring area used for pine (*Pinus sylvestris*). **C**) side view of needle adapters and accessories. **D**) example of assembled large needle leaf adapter (painted black) with needles in measuring area.

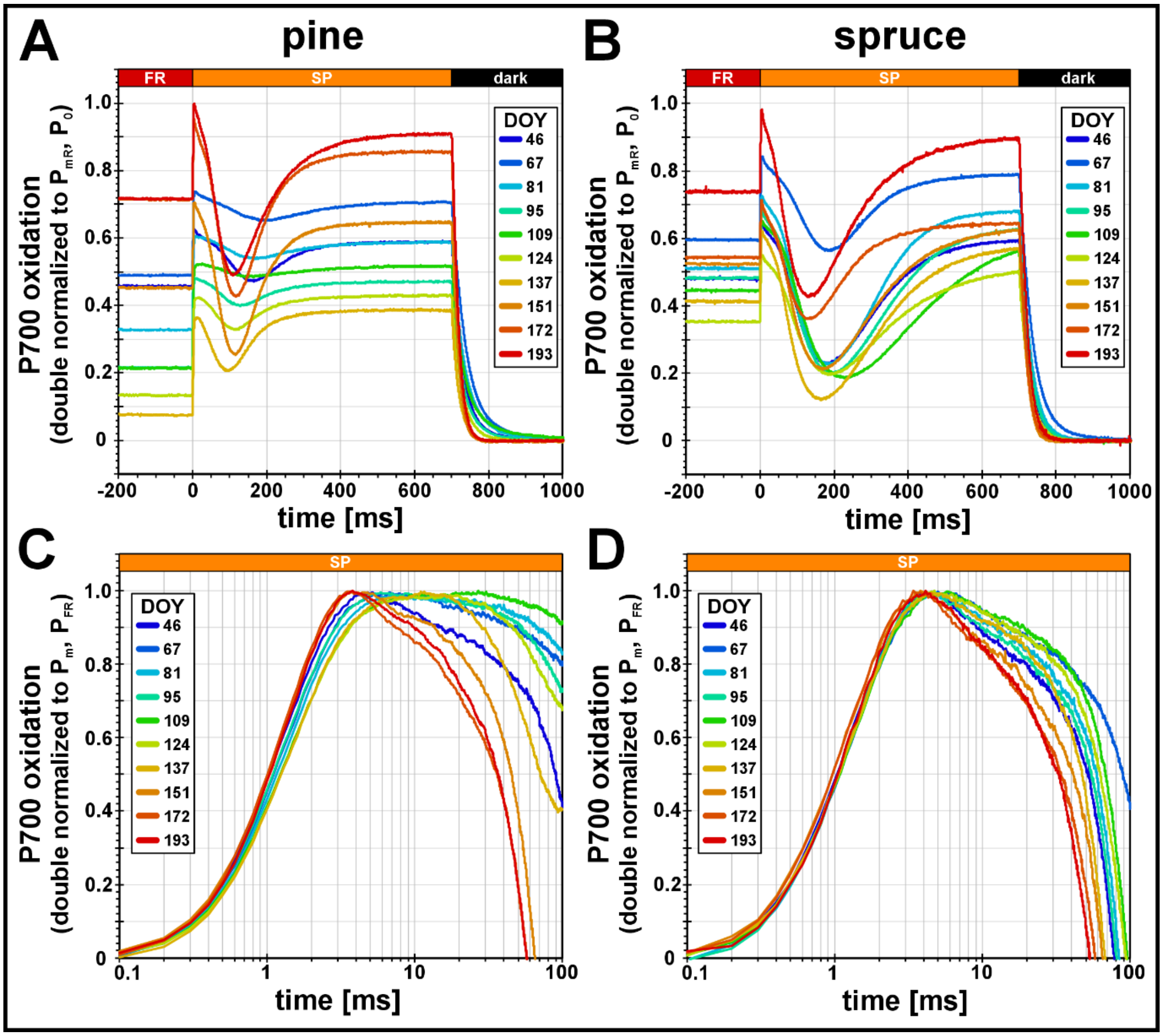

Fig. S2 Seasonal changes in fast-kinetics of maximal P700 oxidation during saturating pulse (ΔP_m determination). (**A-B**) Fast-kinetics of P700 oxidization during ΔP_m determination including far-red pre-illumination (FR, -200-0 ms), saturating pulse (SP, 0-700 ms), darkness (dark, 700-1000 ms) of **A**) pine (*Pinus sylvestris*) and **B**) spruce (*Picea* abies) double normalized to P_{mR} and P_0 absorbance levels. (**C-D**) Fast-kinetics of initial P700 oxidation during SP (first 100 ms) of **C**) pine (*Pinus sylvestris*) and **D**) spruce (*Picea* abies) double normalized to P_m and P_{FR} absorbance levels.

P_{mR} refers to the highest P700 oxidation level (P_m) observed during the season per biological replicate and species, P_{FR} refers to the steady-state P700 oxidation level during FR illumination and P_0 refers to the P700 oxidation level in darkness. All fast-kinetics are the mean of 3 biological replicates per species and sampling point (100 µs resolution). Rainbow colors represent different sampling points (day of year, DOY) from winter (blue) to summer (red).

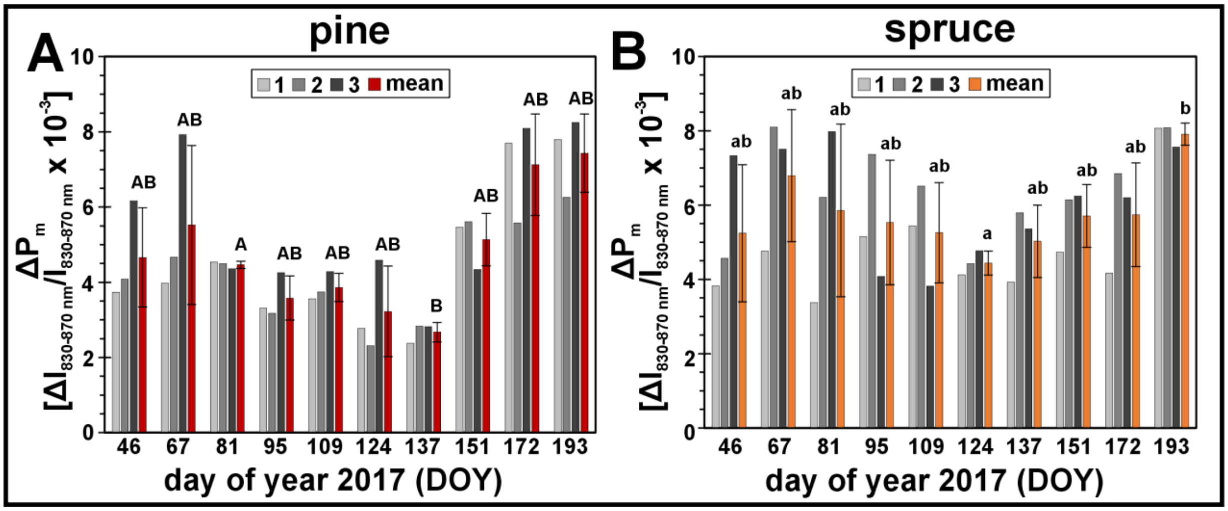

Fig. S3 Seasonal change in maximal redox active PSI fraction (ΔP_m) per biological replicate. Maximal amplitude of P700 difference absorbance signal ($\Delta I_{830\text{-}870\ nm}/I_{830\text{-}870\ nm}$ x 10^{-3}) after far-red pre-illumination (ΔP_m) estimating the maximal redox active PSI fraction in **(A)** pine (*Pinus sylvestris*) and **(B)** spruce (*Picea abies*) throughout the sampling period of 2017. Grey bars represent individual biological replicates (1-3) and colored bars represent mean (red, pine; orange, spruce). Letters represent statistically significant groups (uppercase for pine, lowercase for spruce), which were individually tested per species (Welch's ANOVA, Games–Howell, $p < 0.05$, error bars denote SD, n=3).

Largest ΔP_m per biological replicate and species observed during the sampling period was used as reference ΔP_m (ΔP_{mR}). Pine: biological replicate 1 = DOY 193, biological replicate 2 = DOY 193, biological replicate 3 = DOY 193; Spruce: biological replicate 1 = DOY 193, biological replicate 2 = DOY 67, biological replicate 3 = DOY 81).

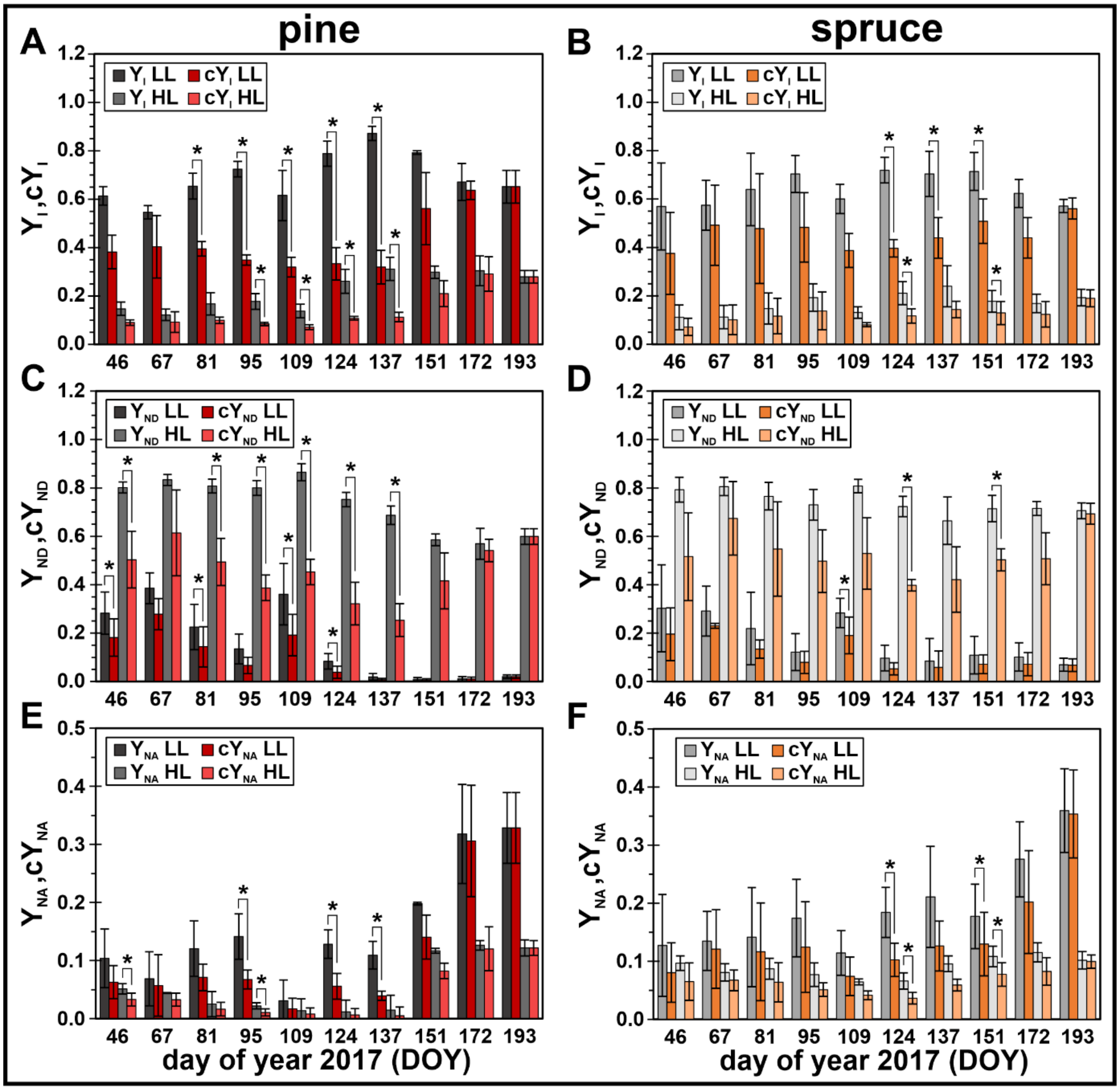

Fig. S4 Overestimation of uncorrected compared to corrected PSI quantum yields. Comparison of classical (uncorrected) and corrected PSI quantum yields in **(A,C,E)** pine (*Pinus sylvestris*) and **(B,D,F)** spruce (*Picea abies*) at representative low light (LL, 100 PAR, dark shading) and high light intensity (HL, 1200 PAR, light shading).

(A-B) Uncorrected (grey) and corrected (pine - red, spruce - orange) yield of effective PSI photochemistry (Y_I, cY_I). **(C-D)** Uncorrected (grey) and corrected (pine - red, spruce - orange) yield of non-photochemical energy dissipation due to PSI donor-side limitation (Y_{ND}, cY_{ND}). **(E-F)** Uncorrected (grey) and corrected (pine - red, spruce - orange) yield of non-photochemical energy dissipation due to PSI acceptor-side limitation (Y_{NA}, cY_{NA}). Error bars denote SD (n=3) with (*) indicating statistically significant differences between uncorrected and corrected PSI quantum yields (paired t-test, $p < 0.05$).

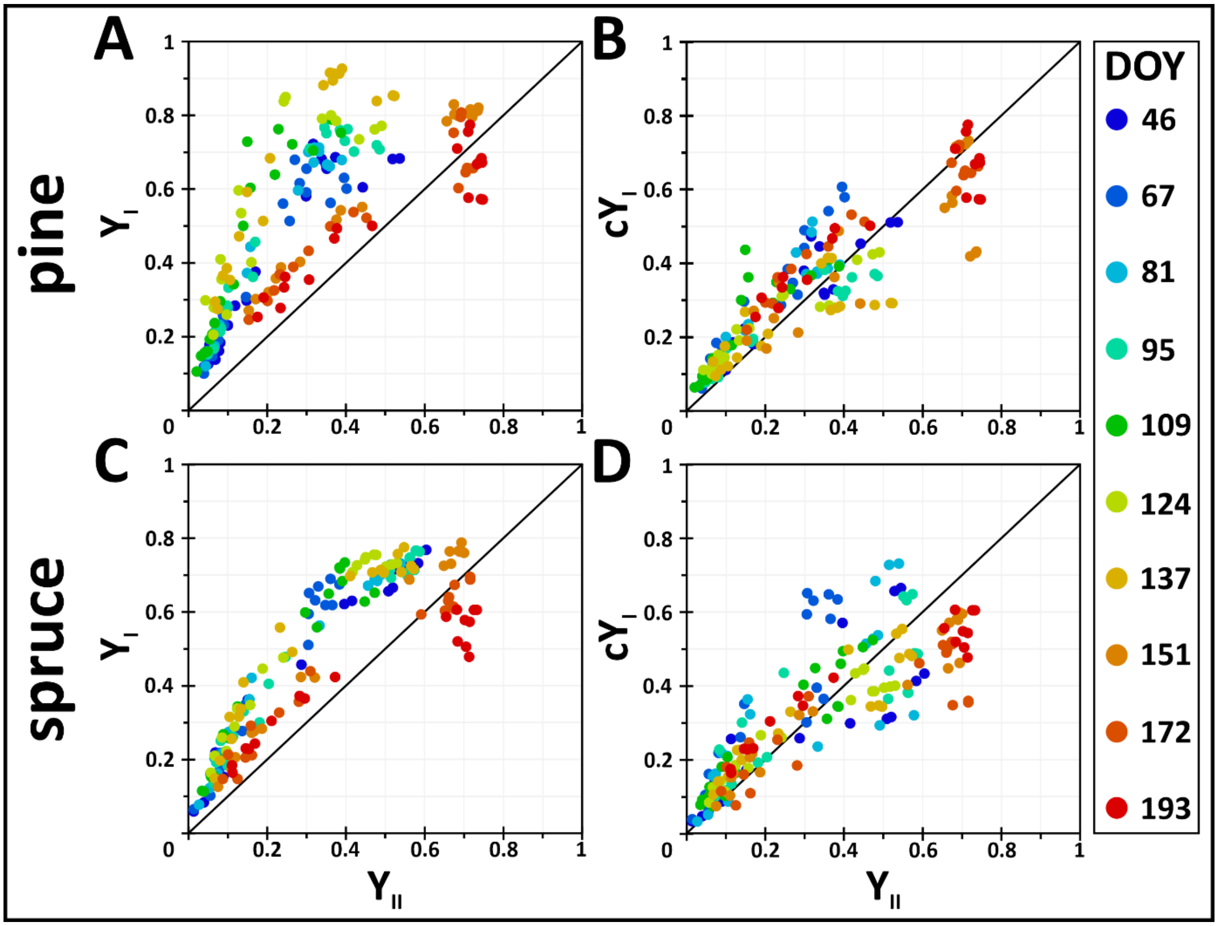

Fig. S5 Seasonal effect of PSI photoinhibition on the functional dynamics of PSI and PSII. Quantum yields of effective photochemistry from PSI and PSII during actinic illumination compared per sampling day (n= 18): **(A)** pine (*Pinus sylvestris*) with uncorrected $Y_I = Y_{II}$, **(B)** pine (*Pinus sylvestris*) with corrected $cY_I = Y_{II}$, **(C)** spruce (*Picea abies*) with uncorrected $Y_I = Y_{II}$, **(D)** spruce (*Picea abies*) with corrected $cY_I = Y_{II}$. Rainbow colors represent different sampling points (day of year, DOY) from winter (blue) to summer (red) with data from all actinic light intensities (25, 50, 100, 400, 800 and 1200 PAR).

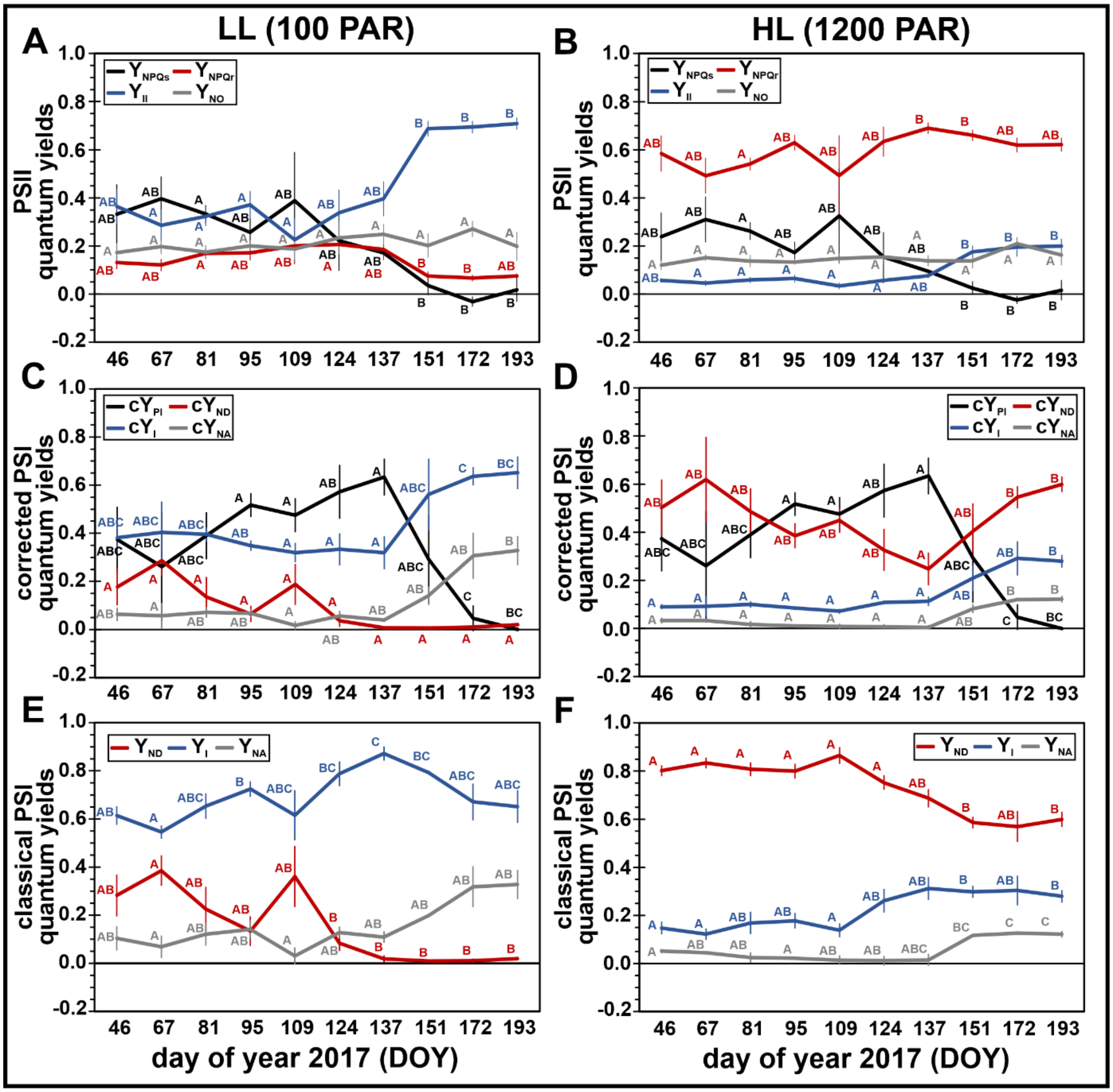

Fig. S6 Seasonal patterns of PSII and PSI quantum yields in pine (*Pinus sylvestris*). PSII and PSI quantum yields from representative **(A,C,E)** low light (LL, 100 PAR) and **(B,D,F)** high light intensity (HL, 1200 PAR). **(A-B)** PSII quantum yields of sustained non-photochemical quenching (Y_{NPQs}, black), reversible non-photochemical quenching (Y_{NPQr}, red), effective PSII photochemistry (Y_{II}, blue) and basal energy dissipation (Y_{NO}, grey). **(C-D)** corrected PSI quantum yields of non-photochemical energy dissipation due to PSI photoinhibition (cY_{PI}, black), non-photochemical energy dissipation due to PSI donor-side limitation (cY_{ND}, red), effective PSI photochemistry (cY_{I}, blue) and non-photochemical energy dissipation due to PSI acceptor-side limitation (cY_{NA}, grey). **(C-D)** classical PSI quantum yields of non-photochemical energy dissipation due to PSI donor-side limitation (cY_{ND}, red), effective PSI photochemistry (cY_{I}, blue) and non-photochemical energy dissipation due to PSI acceptor-side limitation (cY_{NA}, grey). Letters represent statistically significant groups, which were individually tested per parameter (Welch's ANOVA, Games–Howell, $p < 0.05$, error bars denote SD, n=3).

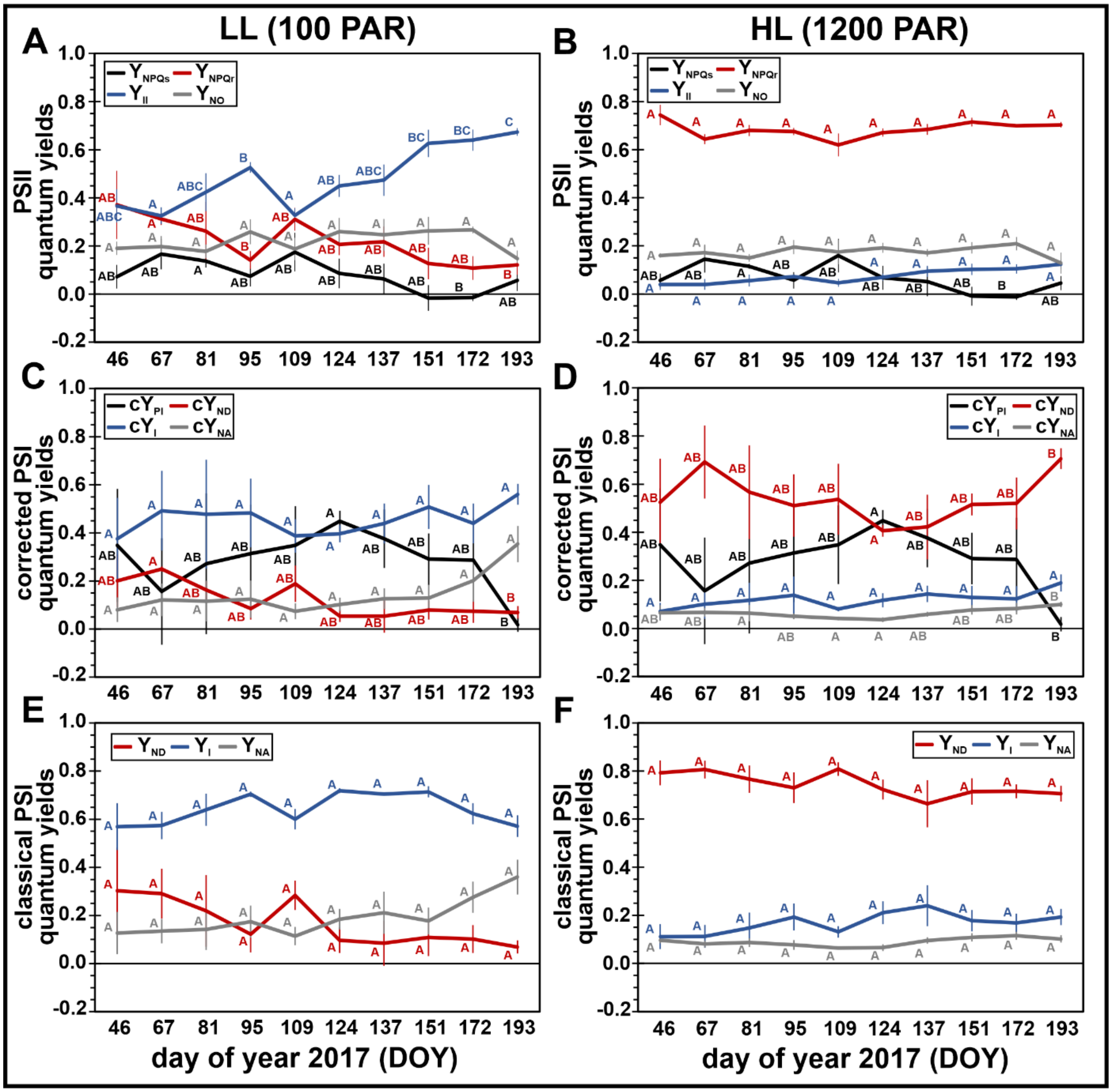

Fig. S7 Seasonal patterns of PSII and PSI quantum yields in spruce (*Picea abies*). PSII and PSI quantum yields from representative **(A,C,E)** low light (LL, 100 PAR) and **(B,D,F)** high light intensity (HL, 1200 PAR). **(A-B)** PSII quantum yields of sustained non-photochemical quenching (Y_{NPQs}, black), reversible non-photochemical quenching (Y_{NPQr}, red), effective PSII photochemistry (Y_{II}, blue) and basal energy dissipation (Y_{NO}, grey). **(C-D)** corrected PSI quantum yields of non-photochemical energy dissipation due to PSI photoinhibition (cY_{PI}, black), non-photochemical energy dissipation due to PSI donor-side limitation (cY_{ND}, red), effective PSI photochemistry (cY_{I}, blue) and non-photochemical energy dissipation due to PSI acceptor-side limitation (cY_{NA}, grey). **(C-D)** classical PSI quantum yields of non-photochemical energy dissipation due to PSI donor-side limitation (cY_{ND}, red), effective PSI photochemistry (cY_{I}, blue) and non-photochemical energy dissipation due to PSI acceptor-side limitation (cY_{NA}, grey). Letters represent statistically significant groups, which were individually tested per parameter (Welch's ANOVA, Games–Howell, $p < 0.05$, error bars denote SD, n=3).

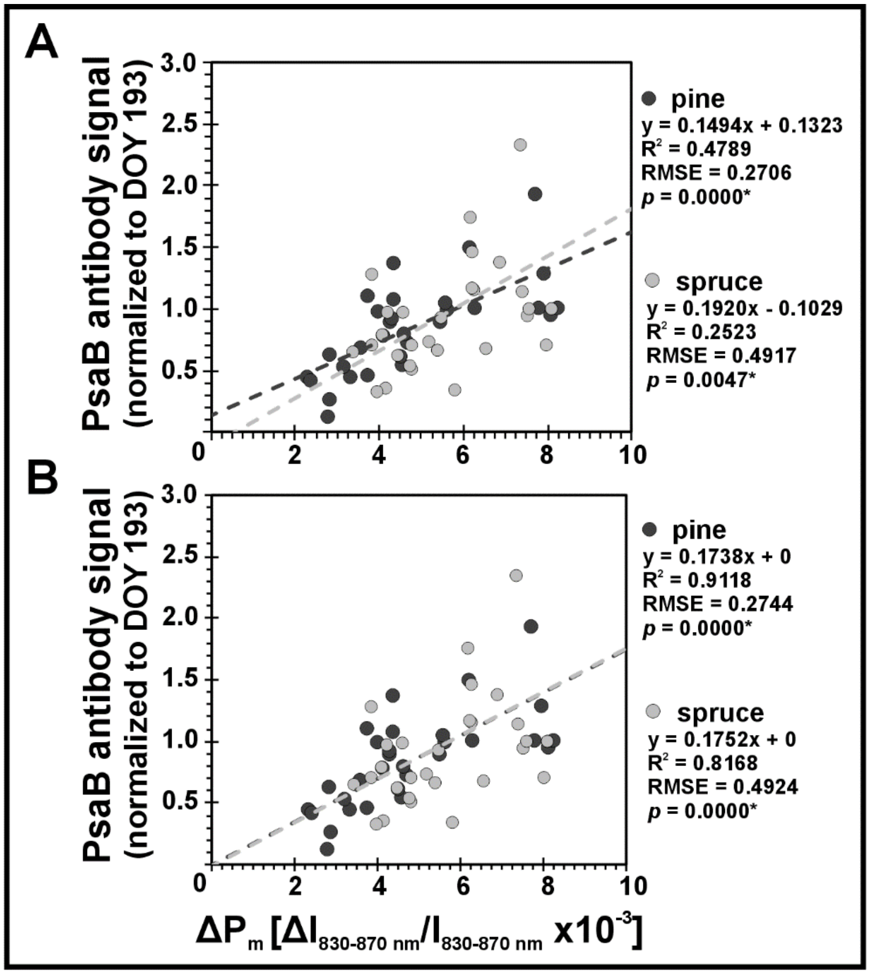

Fig. S8 Linear regression of relative PSI abundance from thylakoid isolations (PsaB) and maximal redox active PSI fraction (ΔP_m) from needle leaves *in-vivo*. **A**) linear regression of PsaB antibody signal (normalized to DOY 193) and ΔP_m without fixed intercept, **B**) linear regression of PsaB antibody signal (normalized to DOY 193) with fixed intercept at the origin. Data (n = 30) of all sampling days (DOY 46 –193) with 3 biological replicates of pine (*Pinus sylvestris* – dark grey) and spruce (*Picea* abies – light grey) were used to calculate coefficient of determination (R^2), root mean square error (RMSE) and significance of regression slope (*p*, unpaired t-test, slope ≠ 0 with (*) indicating $p < 0.05$).

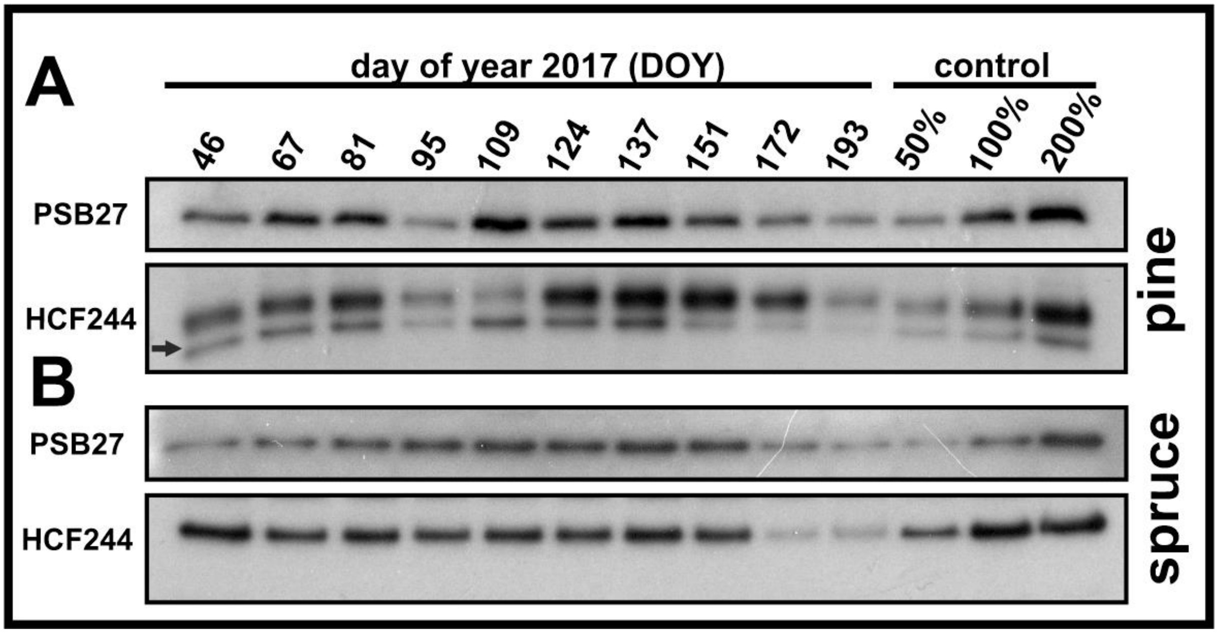

Fig. S9 Seasonal changes in relative abundance of PSII accessory proteins. Representative immunoblots from thylakoid isolations of (**A**) pine (*Pinus sylvestris*) and (**B**) spruce (*Picea abies*) probed with of PSB27 and HCF244 antibodies. In pine, HCF244 is represented by lower band (arrow). Samples were loaded on equal chlorophyll basis. Dilution series in percent of thylakoid samples from DOY 46.
